# Large-scale integrated optoelectronic chaos for machine learning acceleration

**DOI:** 10.1038/s41467-026-73440-2

**Published:** 2026-06-12

**Authors:** Zhouyang Pan, Zhekai Zheng, Ping Li, Hao Wang, Jiacheng Guo, Ding Cui, Zhihui Li, Jiaqi Shen, Lihan Wang, Mengya Zong, Simin Li, Zhe Kang, Yue Yuan, Jianqi Hu, Jijun He, Yuxin Liang, Dan Zhu, Shilong Pan

**Affiliations:** 1 National Key Laboratory of Microwave Photonics, Nanjing University of Aeronautics and Astronautics, Nanjing, China; 2 Department of Precision Instrument, Tsinghua University, Beijing, China; 3 United Microelectronics Center, Chongqing, China; 4 Department of Cardiology, The First Affiliated Hospital with Nanjing Medical University, Nanjing 210029, China; 5 Department of Electrical and Electronic Engineering, The University of Hong Kong, Hong Kong, China

**Keywords:** Integrated optics, Microwave photonics, Nonlinear optics, Frequency combs

## Abstract

Chaos has emerged as a useful resource for machine learning, yet traditional nonlinear circuits face speed bottlenecks. Optical chaos sources offer an attractive alternative with ultra-wideband operation and massive parallelism, but existing schemes must trade single-channel throughput against multi-channel scalability. Here, we demonstrate an integrated microcomb-optoelectronic chaos engine (iMOCE). By driving an optoelectronic nonlinear cavity with a chaotic microcomb, the iMOCE generates massively parallel chaos with a 6-dB bandwidth of 25 GHz per channel, representing a two-order-of-magnitude improvement over previous microcomb-based approaches. The system delivers a total random-bit generation rate of 32.768 Tbps and accelerates four representative tasks. Compared with MCU/GPU baselines, it reduces per-inference time by about two orders of magnitude. These results establish iMOCE as a scalable, massively parallel chaos primitive for machine learning acceleration.

## Abstract (web summary)

Researchers demonstrate an integrated optoelectronic chaos engine that generates massively parallel, ultra-wideband randomness and accelerates machine-learning tasks by about two orders of magnitude, offering a promising route to probabilistic computing.

## Introduction

Chaos, arising from the deterministic dynamics, exhibits aperiodic and unpredictable behavior ^1,2^. The random and unpredictable characteristics of chaos have found increasing utility in the areas of machine learning (ML) acceleration ^3–10^, private communication ^11–13^, and LiDAR ^14,15^. Especially in ML, chaos serves as high-quality pseudo-random perturbations for reinforcement learning (RL) and genetic algorithms ^5–7^. It also serves as a regularizer during neural-network training, enhancing the classification performance ^4^. Digital pseudo-random and digital-chaos sources built on nonlinear analog circuits are widely used, but the throughput and processing time are constrained by the electronic bandwidth. To date, the frequency of chaos rarely exceeds a few GHz, and scaling throughput requires massive and parallel arrays, severely restricting potential acceleration performance and system integration ^16–18^.

Optical chaotic sources provide an attractive alternative, offering advantages of ultra-wideband, inherent nonlinearity, and massive parallelism. Techniques based on chaotic lasers ^19–26^, optomechanical chaotic resonators ^27–31^, and optoelectronic nonlinear cavities (OENC) ^32–35^ have demonstrated high-speed chaos generation, but each relies on a single feedback/coupling loop. Therefore, multi-channel independent chaos generation requires replicating entire setups (e.g., lasers, cavities, or delay lines), inevitably increasing the system complexity, power consumption, and cost. As such, scalable optical chaos sources based on fully integrated photonic platforms remain a challenge.

Integrated microring resonator (MRR)-based chaotic microcombs offer a promising scalable solution by leveraging wavelength-division multiplexing (WDM) of comb lines, yielding multiple broadband and uncorrelated chaos on a single chip ^36–40^. Their compact footprint, low power consumption, and native parallelism make them suitable for high-throughput optoelectronic accelerators. However, their spectral uniformity is limited by relaxation oscillations, which generally have a limited 6-dB bandwidth below GHz per channel ^40^. Moreover, their power imbalance requires complex post-processing for equalization. Decreasing the optical quality factor (Q) can broaden the bandwidth, but at the cost of higher pump power and degraded chaotic quality ^41–43^. Such a tradeoff critically constrains the acceleration speed and the response time of large-scale, high-speed ML workloads using chaotic microcombs. In addition, chaotic microcombs have so far been applied mainly to random bit generation and small-scale multi-armed bandit (MAB) acceleration problems, leaving complex ML tasks, such as chess games ^44–46^, global combinatorial optimization problems ^47–49^, and class recognition tasks ^50–52^ unexplored.

**Fig. 1:**
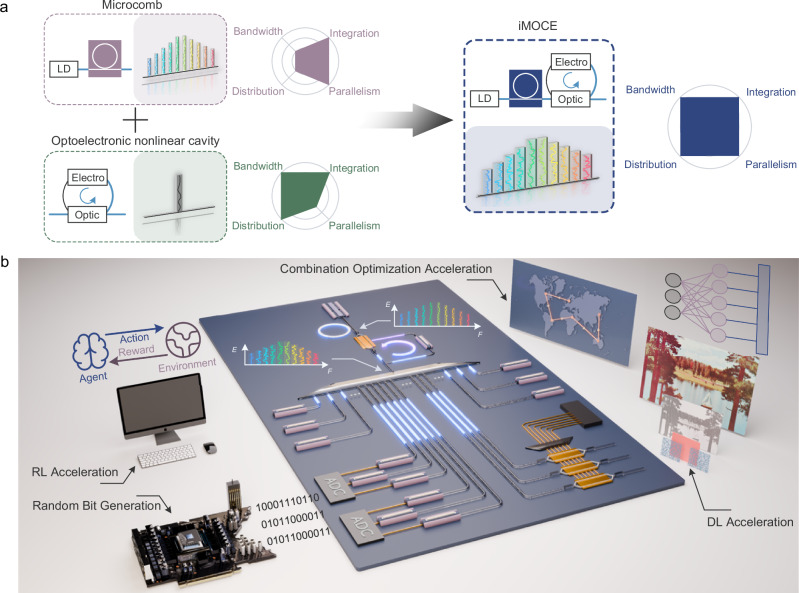
Concept and application scope of the iMOCE. **a** The iMOCE combines the high parallelism of a microcomb with the large bandwidth of an optoelectronic nonlinear cavity to generate parallel, ultra-wideband chaos with a favorable probabilistic distribution and without post-processing. **b** Schematic of optical chaotic sources based on the iMOCE and their applications in machine-learning acceleration, random-bit generation, combinatorial-optimization acceleration, and deep-learning acceleration.

In this work, an integrated microcomb-optoelectronic chaos engine (iMOCE) is proposed to overcome these bottlenecks. Figure 1 illustrates the concept and application scope of the iMOCE. The iMOCE combines the advantages of high parallelism of microcomb and large bandwidth of the OENC to generate parallel, ultra-wideband chaos with a favorable probabilistic distribution without post-processing. Notably, the chip, fabricated in a commercial foundry and compatible with wafer-scale production, extends the per-channel 6-dB bandwidth to 25 GHz (two orders of magnitude beyond prior microcomb schemes). The feasibility of the multi-channel large-bandwidth chaotic entropy sources is verified by benchmarking five representative tasks, including random bit generation, MAB, connect-3, traveling-salesman solving, and electrocardiogram (ECG) trace recognition. By integrating optical chaos on a wafer-scale integrated photonic platform, iMOCE functions as a robust computing resource that provides a scalable, massively parallel physical entropy source for heterogeneous ML workloads and is poised for system-level deployment.

## Results

### Features of the generated parallel and ultra-wideband chaos

**Fig. 2:**
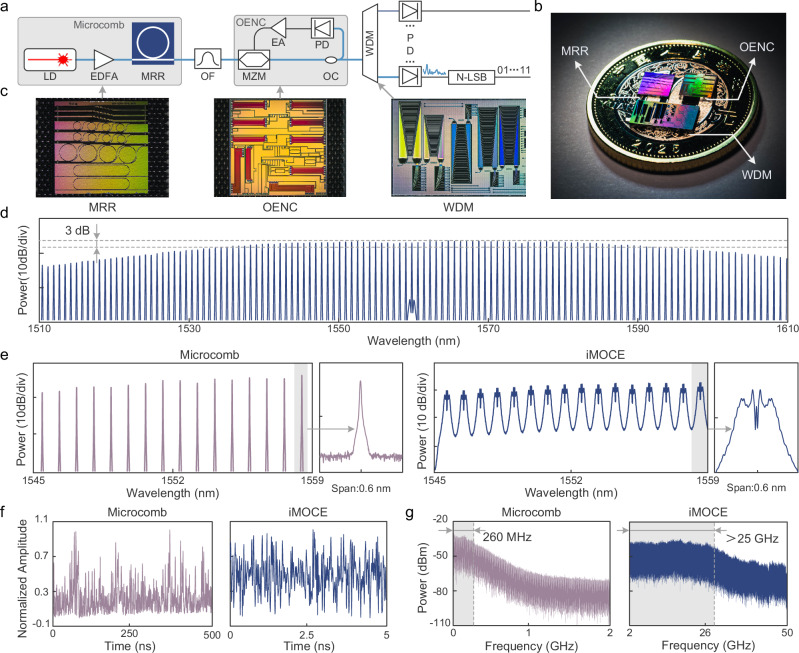
Integrated architecture and generated chaos of the iMOCE. **a** The setup of the realized iMOCE architecture. LD, laser diode; EDFA, erbium-doped fiber amplifier; MRR, microring resonator; OF, optical filter; MZM, Mach-Zehnder modulator; OC, optical coupler; PD, photodetector; EA, electrical amplifier; WDM, wavelength-division multiplexing; LSB, least significant bits. **b** Microscope image of the chip. **c** Optical microscope image of the MRR, OENC, and WDM. **d** Optical spectrum of the generated chaotic comb. **e** Optical spectra of the selected microcomb and the selected iMOCE, respectively. **f** Time-domain waveform of the generated chaos, recorded by the real-time oscilloscope. **g** Frequency-domain waveform of the generated chaos, recorded by the electrical spectrum analyzer. With the OENC engaged, the 6-dB bandwidth increases from 260 MHz to 25 GHz, corresponding to a two-orders-of-magnitude expansion.

Figure 2a illustrates the schematic diagram of the realized iMOCE architecture, comprising microcomb, OENC, parallel channel separation, and digital readout. First, a tunable semiconductor laser, amplified by an erbium-doped fiber amplifier (EDFA), is coupled into an MRR. By adjusting the MRR temperature with a thermo-electric controller (TEC), the pump wavelength is swept toward the resonance wavelength. As the pump resonance detuning decreases, the spectrum evolves from a primary comb to a sub-comb and finally to a broadband chaotic comb. Figure 2d presents the chaotic comb spectrum generated at the temperature of 36.6 ^∘^C, showing about 150 lines in the wavelength range between 1500 nm and 1600 nm. An optical filter (OF) then selects sixteen comb lines, as shown in Fig. 2e. Importantly, the output of the OF is injected into an OENC, comprising a Mach-Zehnder modulator (MZM), an optical coupler (OC), a photodetector (PD), and an electrical amplifier (EA).

The resulting spectral broadening of the OENC output is presented in Fig. 2e. Consequently, a WDM receiver employs a 16-channel arrayed-waveguide grating (AWG) that, upon injection of the amplified comb, selectively demultiplexes target lines and routes them to the PD array. For comparison, the chaotic comb before the OENC is routed through the same separation stage. More details about chips can be found in the Supplementary Note 1.

**Fig. 3:**
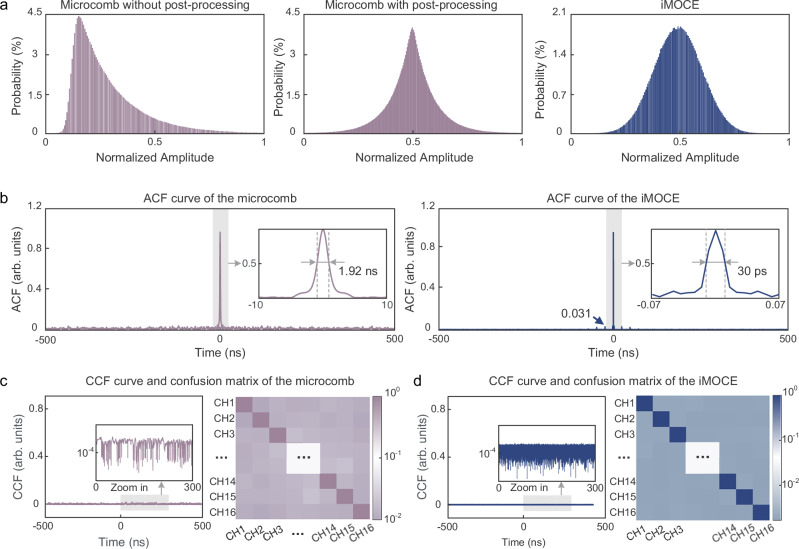
Statistical characteristics of the generated parallel chaos. **a** Amplitude distributions of the chaos. The raw microcomb shows asymmetric histograms that typically require post-processing, such as delay-difference, self-delayed XOR, or wavelet transforms. By contrast, the iMOCE output is intrinsically symmetric, removing the need for such correction. **b** Auto-correlation-function curves of the chaos. The full width at half maximum decreases from 1.92 ns to 30 ps, consistent with the measured 16-dB bandwidth of the chaos. **c** Cross-correlation-function curves and confusion matrix between comb lines. Pairwise inter-channel correlations remain below 0.047 for the off-diagonal entries, confirming low mutual dependence and suitability for parallel operation.

To verify the performance of the proposed architecture as a massively parallel chaos source, the time-domain and frequency-domain characteristics of the generated chaos based on the microcomb and the iMOCE are presented in Fig. 2f, g, respectively. By incorporating the OENC, the 6-dB bandwidth of the chaos expands substantially from 260 MHz to 25 GHz. The amplitude distribution of the raw microcomb exhibits asymmetric histograms that typically require complex post-processing (e.g., delay-difference, self-delayed XOR, or wavelet transforms) for correction. After being injected into the OENC, the photodetector converts the unipolar optical intensity fluctuations into an electrical current. The subsequent bandpass amplification chain effectively removes the DC component. As a result, the output of the iMOCE is inherently symmetric. This bypasses the complex post-processing steps, making it highly favorable for direct application in ML acceleration, as shown in Fig. 3a.

To evaluate the chaotic characteristics of intra-channel and inter-channel, the autocorrelation function (ACF) curves and the cross-correlation function (CCF) curves are presented in Fig. 3b, c. The full-width at half-maximum (FWHM) of the ACF curves shrinks from 1.92 ns to 30 ps, corresponding to the 16-dB bandwidth of chaos. As shown in the ACF curve of the iMOCE, a secondary ACF peak with a time location of 24 ns and an intensity of 0.031 matches the loop delay. As shown in Fig. 3c, inter-channel correlations remain below 0.047, confirming the suitability of the proposed chaotic source for parallel operation.

### Random bit generation

**Fig. 4:**
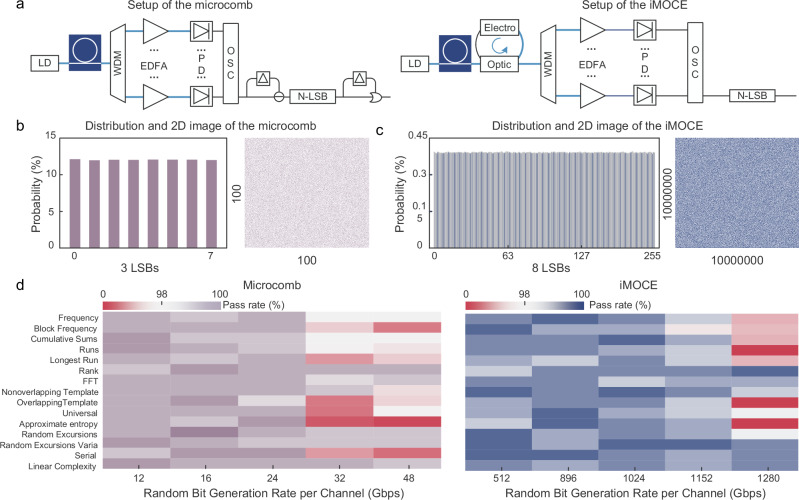
Parallel random-bit generation enabled by the iMOCE. **a** Setup of the parallel random-bit generation task. The iMOCE output exhibits an inherently uniform and symmetric amplitude distribution, requiring neither down-sampling nor complex post-processing before bit extraction. OSC, real-time oscilloscope. **b**,** c** Distribution of the extracted *N* LSBs and the two-dimensional image of the first 1 M-bit sequence generated by the system. Histograms of the extracted LSBs per sample show near-flat distributions, and two-dimensional bit maps confirm spatial uniformity and randomness. **d** Results of the NIST tests as the random-bit generation rate increases. Both sources satisfy the randomness criteria up to their per-channel limits, namely 24 Gb/s for the microcomb and 1.024 Tb/s for the iMOCE, beyond which the *p*-values drop below 0.0001, or the pass rates fall below 0.98045.

To evaluate the feasibility of using the iMOCE architecture as a high-throughput parallel chaotic source, a random bit generation process is implemented, as illustrated in Fig. 4a. The generated chaos sequences are captured by a real-time oscilloscope (OSC) with a sampling rate of 128 GSa/s and a resolution of 12 bits. c For the microcomb configuration, signals are down-sampled to 8 GSa/s to reduce inter-sample correlation, and then passed through a delay-difference filter to symmetrize the amplitude distribution^37^. The result is quantized to 12 bits, with the 3 least significant bits (LSBs) extracted and a final XOR operation applied to remove residual bias. In contrast, the output of the realized iMOCE architecture exhibits inherently uniform and symmetric amplitude distribution, eliminating either down-sampling or complex post-processing. In addition, 8 LSBs are harvested directly, yielding a dramatic increase in throughput and a reduction in latency. Their amplitude histograms (Fig. 4b, c) display near-flat distributions, and two-dimensional bit-map plots of the first 10^6^ bits (-1’ = white, -0’ = purple/blue) confirm spatial uniformity and randomness.

Statistical unpredictability is assessed via the NIST SP 800-22 test suite on 1000 sequences of 10^6^ bits each at a significance level of *α* = 0.01, as shown in Fig. 4d. Both systems meet randomness criteria only up to their respective per-channel rates-24 Gbps for the microcomb and 1024 Gbps for the iMOCE, beyond which p-values will fall below 0.0001, or pass rates will dip under 0.98045.

Overall, by using the iMOCE architecture, the bit rate of chaotic combs increases from 768 Gbps (32 channels × 8-GSa/s sampling rate × 3-bit LSB) to 32.768 Tbps (32 channels × 128-GSa/s sampling rate × 8-bit LSB). Table 1 lists the comparison between this work and existing schemes, showing that the iMOCE achieves the highest generation rate and bandwidth among photonic chaos methods.

**Table 1:** Comparison of the proposed work with published parallel random-bit generation schemes based on optoelectronic chaotic sources

Scheme	Bandwidth (GHz)	Total bit rate (Gbps)	Single-channel rate (Gbps)	Channel number
Amplified spontaneous emission^55^	12	20	10	2
Quantum vacuum state^56^	/	3.08	0.44	7
Optical super-continuum^57^	/	40	10	4
Chaotic microcomb^36^	0.3	2240	320	7
Chaotic microcomb^37^	1	3840	120	32
Chaotic OENC^58^	/	1.478	1.478	1
Chaotic OENC^59^	15	160	80	2
Chaotic OENC^60^	8	250	250	1
Chaotic OENC^61^	9.19	500	500	1
Chaotic OENC^62^	12	2500	1280	2
**This work**	**25**	**32,768**	**1024**	**32**

### Connect-3 game acceleration

**Fig. 5:**
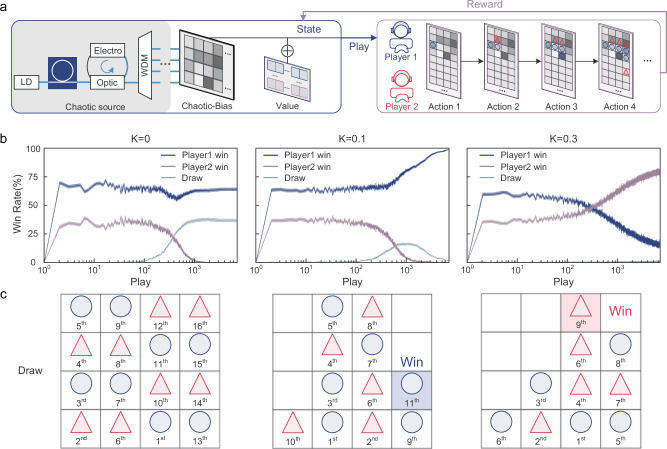
Connect-3 game using chaotic bias. **a** Setup of connect-3 game acceleration based on the iMOCE. Chaos from 16 channels forms a 4 × 16 chaotic-bias matrix, where each row maps to a board position and each column to a move index. The cumulative bias, scaled by coefficient *K*, is added to the value matrix to yield the current-state matrix. The maximum-value entry dictates each player’s move. **b** Win rates and **c** decision-making results for the 5000^*th*^ play under different chaotic intensities. With pure greedy play, the first-move advantage gives a peak win rate of 70%, but convergence often stalls at suboptimal policies. At moderate intensity, enhanced exploration accelerates convergence to the optimal policy and reaches a 100% win rate. With excessive randomness, strategy coherence degrades and the win rate drops.

To evaluate the efficacy of the realized iMOCE architecture in accelerating decision-making within zero-sum games, a connect-3 game built on a 4 × 4 chessboard is conducted. In the connect-3 game, two players alternately place tokens, starting from the bottom of a chosen column. The decision-making process is illustrated in Fig. 5a. In this process, chaos generated by the 16 channels of the iMOCE architecture is set to a host PC via a gigabit Ethernet interface, where a Python pipeline performs online processing. The received chaos constructs a 4 × 16 chaotic-bias matrix, where each row maps to a board position and each column to a move index. This bias matrix is cumulatively added to a value matrix to yield the current state matrix, and the maximum-value entry dictates each player’s move. The influence of the chaotic bias matrix on the state matrix is determined by the coefficient *K*. After each game, the outcome updates the value matrix via the 1^*st*^ temporal-difference learning method. Notably, board updates, win detection, and the time-ordered log of visited states are all implemented digitally.

To further demonstrate the role of chaos in accelerating the game and optimizing convergence, player 1 received chaotic bias in addition to a greedy policy, while player 2 used only the greedy policy. Figure 5b, c traces the winning rates and decision-making outcomes over 1000 independent trials, with each trial consisting of 5000 sequential plays. The shaded regions indicate the standard error of the mean (SEM) across 1000 independent trials. In each trial, the value function is initialized as 0, and the value matrix is updated after each play. While the algorithm and hyperparameters remain constant across all 1000 trials, the policy in each trial samples data from a distinct time segment of the iMOCE stream, yielding unique exploration trajectories. As shown in Fig. 5b, c, with pure greedy play (*K* = 0), the first-move advantage of player 1 yields a peak win rate of about 70%, but convergence often stalls in suboptimal strategies. As shown in Fig. 5c, a winning placement at position (3,3) on the 8^*th*^ move is bypassed due to convergence on a suboptimal strategy, resulting in a draw result. At moderate chaos intensity (*K* = 0.1), exploration of superior move sequences accelerates convergence to an optimal policy and achieves a 100% win rate. Beyond this optimal range (*K* = 0.3), excessive randomness degrades strategy coherence and reduces the win rate.

### Heart arrhythmia recognition acceleration

**Fig. 6:**
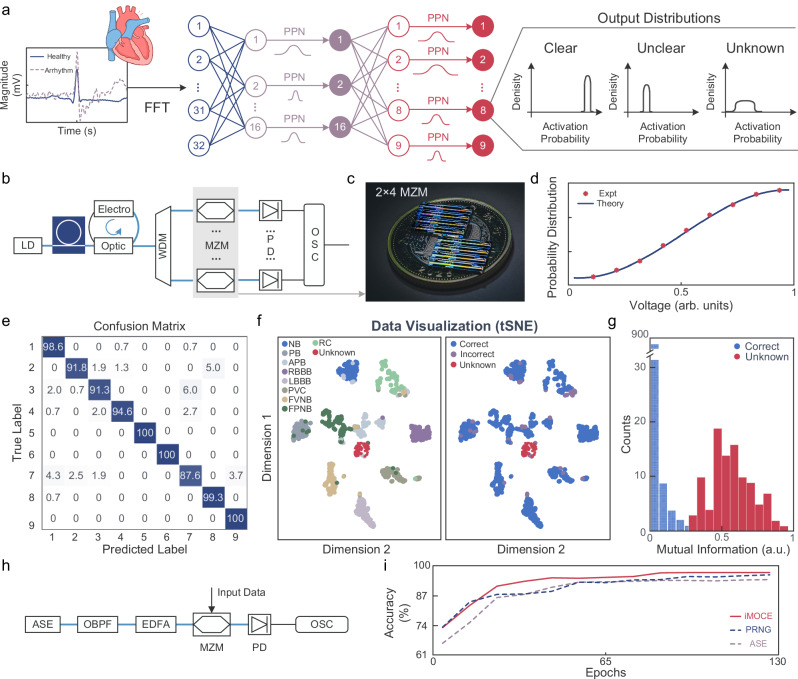
Heart arrhythmia recognition with a photonic probabilistic neural network. **a** Setup of heart-arrhythmia-recognition acceleration based on the iMOCE. Each ECG trace is converted into a 32-point vector through a fast Fourier transform (FFT) and fed to a two-layer probabilistic neural network. **b** Schematic of the photonic probability neuron (PPN). The iMOCE supplies statistically independent chaotic samples through the outputs of the last layer to realize variational posterior sampling. After training, the network outputs a posterior class-probability vector for each input, from which the predicted label and epistemic uncertainty are obtained. **c** Microscope image of the MZM array. **d** Probability distributions of the generated chaos as the applied voltage is changed. **e** Confusion matrix of the recognition model, which reaches an accuracy of 95.7%. **f** t-SNE visualization of the input data, where nearby points correspond to similar samples and distant points correspond to dissimilar samples. **g** Mutual-information distributions of the correctly classified and unseen data. The two distributions show clear separation, demonstrating successful rejection of out-of-distribution inputs. **h** Experimental setup of the amplified spontaneous emission (ASE)-noise-based optical noise source. **i** Validation accuracy as a function of training epoch when driven by three different random sources.

To evaluate the ability of the iMOCE architecture in accelerating the class recognition task, a probabilistic neural network is constructed to discriminate nine clinically relevant arrhythmia categories from ECG traces ^53^. During the inference process, a tenth “unknown” class is injected to probe the out-of-distribution (OOD) mode. As illustrated in Fig. 6a–c, each recorded ECG trace is converted into a 32-point vector through a Fast-Fourier Transform (FFT) and presented to a two-layer probabilistic neural network comprising 32 inputs, 16 hidden units, and 9 output neurons. Notably, the weights are trained as point estimates, while photonics probability neurons (PPN) realized by the iMOCE supply statistically independent chaotic samples injected at layer activations. Repeating the forward pass for the same input yields Monte Carlo samples of a random function, from which both the predicted label and its epistemic uncertainty are extracted. As shown in Fig. 6a, for a clear prediction, all posterior weight samples collapse onto a single class, thereby enabling the activation probability of one output neuron to approach 1. For an unclear prediction, the measurement noise broadens the posterior such that several networks vacillate between the same pair of classes, enabling the activation probability of one output neuron to approach 0.5. For an unknown prediction, all neural networks sampled from the probabilistic neural network will tend to make a different interpretation of this unknown data, resulting in a high variance in the distribution of output neurons. Adam is employed as the optimizer, and the initial learning rate is set to 0.01 without decay. The batch size is set to 32, and the model is trained for 200 epochs. Notably, because the probability output of the integrated PPN depends on the bias applied to the MZM array, the statistical envelopes measured at the photodetector vary accordingly, as shown in Fig. 6d.

The confusion matrix in Fig. 6e confirms an overall classification accuracy of 95.7%, while Fig. 6f shows the per-class performance in detail. To visualize the recognition performance, a t-distributed stochastic neighbor embedding (t-SNE) method is applied. This visualization method maps each high-dimensional sample to a single point in a two-dimensional space such that similar samples cluster closely together, whereas dissimilar ones are projected far apart. As shown in Fig. 6f, incorrect recognition points cluster near the boundaries of major aggregates, indicating intrinsically conflicting examples. Finally, the mutual-information map in Fig. 6g reveals a pronounced separation between correct and unknown samples, demonstrating successful rejection of OOD inputs.

To test the performance of the ECG task under different random sources, the probability training is repeated while the iMOCE chaos is replaced by either a digital pseudo-random number generator (PRNG) or an amplified spontaneous emission (ASE) noise-based optical white-noise source (Fig. 6h). As shown in Fig. 6i, all three random sources produce nearly identical learning curves and converge to final accuracy of 95.5%, 94.6%, and 92.3%, respectively. This confirms that the iMOCE acts as a high-quality physical random engine that can replace conventional noise sources without degrading accuracy.

## Discussion

Compared with prior photonic chaotic sources based on microcombs, the iMOCE architecture delivers more than a two-order-of-magnitude increase in single-channel bandwidth. The architecture also produces chaos with inherently symmetric amplitude distributions without the need for complex post-processing. Further performance gains are attainable by (1) adopting wide-band electro-optics devices to extend per-channel bandwidth, (2) increasing the number of WDM and ADC channels to exploit additional comb lines, and (3) engineering MRR dispersion to broaden the spectral coverage.

In addition, a notable strength of this approach is integration. The heterogeneous integration between III-V/Si lasers with ultralow-loss SiN MRR on a silicon platform has been demonstrated for on-chip soliton microcombs. The self-injection locking of a DFB laser to a nonlinear MRR has been shown to enable stable and chip-scale soliton microcomb operation. In such heterogeneous integration, fiber-to-chip coupling losses are significantly reduced, and a bulky EDFA is often not required. When optical gain is still required, the on-chip semiconductor optical amplifier (SOA) or an erbium-doped waveguide amplifier (EDWA) can be introduced, which primarily reduces system volume and packaging complexity while improving end-to-end efficiency. The resulting photonic accelerator can interface directly with dedicated electronic integrated circuits. This integration minimizes footprint, power consumption, and cost while enhancing the computational throughput beyond the conventional semiconductor technology limits.

Finally, this work constitutes the first demonstration of an optoelectronic chaotic source applied across four benchmark ML tasks. The tasks cover the high-speed random bit generation, the 1024-armed bandit problem, the adaptive connect-3 game, the Hopfield-network-based traveling-salesman problem, and the heart arrhythmia recognition. Measured outcomes show a reduction in convergence cycles from 12,650 to 8200 (MAB, see Supplementary Note 2), an increase in win rate from 70% to 100% (connect-3), a shortening of the best tour from 5.5363 m to 3.3593 m (traveling-salesman solving, see Supplementary Note 3), and 95.7% accuracy with reliable OOD rejection (ECG). Each task demonstrates that iMOCE provides high-quality physical entropy comparable to ideal sources, while enabling distinct hardware advantages in throughput and integration. Table 2 compares these results with leading optical accelerators, highlighting the superior performance of the realized iMOCE architecture. Notably, the iMOCE can be further employed in cryptography where a more conservative extraction configuration is required to obtain stronger guarantees and higher min-entropy bounds. Because iMOCE has Tb/s-class physical bandwidth, it can further lower the sampling rate, reduce the number of bits extracted per sample, and adopt stronger post-processing to trade throughput for larger entropy margins and lower correlation.

**Table 2:** Comparison with state-of-the-art photonic machine-learning accelerators

Scheme	Bandwidth (GHz)	Channel number	ML tasks	External equipment
Chaotic laser^63^	1	2	2-armed bandit problem	Yes
Chaotic laser^64^	2	1	1024-armed bandit problem	Yes
Chaotic laser^65^	/	/	512-armed bandit problem	Yes
Chaotic laser^66^	/	/	512-armed bandit problem	Yes
Chaotic laser^67^	30	4	16-armed bandit problem	Yes
Chaotic microcomb^37^	1	32	256-armed bandit problem	No
Chaotic microcomb^39^	0.75	5	512-armed bandit problem	No
Chaotic OENC^32^	18	4	512-armed bandit problem and chess (Tic-Tac-Toe) game	Yes
**This work**	**25**	**32**	**1024-armed bandit problem, chess (connect-3) game, Hopfield-network-based traveling-salesman problem, and heart arrhythmia recognition task**	**No**

Although current digital calculation and judgment in the work impose a bottleneck on decision-making latency, and the advantages of the optoelectronic accelerator in terms of speed are not fully used in the current situation, various potential applications of fast acceleration can still be expected. For instance, next-generation mobile networks will require millisecond or sub-millisecond slice allocation and QoS management for VR/AR and vehicle-to-everything services, while autonomous vehicles and drone swarms demand collision warnings on the order of milliseconds ^54^.

As such systems continue to evolve toward higher parallelism, shorter decision cycles, and stronger local security requirements, the proposed optical chaotic source can further improve throughput, real-time performance, energy efficiency, and physical unpredictability. Furthermore, by combining the optical chaos source with optical or optoelectronic operators, such as weight kernels, nonlinearities, and comparators, stochastic computation and decision-making can be performed primarily at the front end ^4^. As such, the GPU/CPU will receive only highly compressed decisions or a small set of statistics, which significantly accelerates the speed of decision-making. Fast optoelectronic ML accelerators such as our iMOCE thus hold promise for these real-time systems.

## Methods

### Experimental setup

In the experiment, a continuous-wave light with a wavelength of 1550.3 nm is provided by a laser (TOPTICA CTL 1550, 160-kHz linewidth) and amplified to 25 dBm by an EDFA (CONNET MFAS-EY-C-B) to serve as the pump. The pump is coupled into a silicon nitride MRR with a radius of 227 μm and a waveguide cross-section scale of 0.8 × 2 μm, yielding a free spectral range of approximately 100 GHz and a loaded quality factor of 7 × 10^6^. The temperature of the MRR is stabilized at 23 ± 0.1 ^∘^C using a TEC. Subsequently, multi-channel chaos is generated, and its spectrum is recorded by a spectrometer (Yokogawa AQ6370D) with a resolution of 0.02 nm and a span of 1200 nm. An optical filter (Finisar 4000S) is then employed to select sixteen comb lines, which are then directed into an integrated MZM with a working bandwidth of 30 GHz. The MZM output is detected by an on-chip PD with a working bandwidth of 30 GHz, amplified electrically, and fed back to the MZM to form the OENC structure. The resulting multi-channel broadband chaotic output is spread by a WDM, and each channel is captured by a PD. The time domain of the obtained signal is acquired by an OSA(Keysight UXR3304B) with a sampling rate of 128 GSa/s, while the spectra are measured by an electrical spectrum analyzer (ESA, Keysight N9020B) with a resolution bandwidth of 1 MHz and a span of 50 GHz.

### Characterization of the generated optical chaos

The optical chaos generated by the iMOCE is captured by the OSA and subjected to the correlation analysis. For channel *i* with discrete temporal sequence *x*_*i*_, the normalized auto-correlation and cross-correlation function are mathematically calculated as

1 $$AC{F}_{i}(\tau )=\left| \frac{{\sum }_{n=1}^{N}[{x}_{i}(n)-{x}_{i0}]\cdot [{x}_{i}(n+\tau )-{x}_{i0}]}{{\sum }_{n=1}^{N}{[{x}_{i}(n)-{x}_{i0}]}^{2}}\right|$$

2 $$CC{F}_{i,j}(\tau )=\left| \frac{{\sum }_{n=1}^{N}[{x}_{i}(n)-{x}_{i0}]\cdot [{x}_{j}(n+\tau )-{x}_{j0}]}{\sqrt{{\sum }_{n=1}^{N}{[{x}_{i}(n)-{x}_{i0}]}^{2}\cdot {\sum }_{n=1}^{N}{[{x}_{j}(n)-{x}_{j0}]}^{2}}}\right|$$

where *x*_0_ is the average value.

### Connect-3 game

The rewarding process of the connect-3 game follows the temporal-difference learning method, which is given as

3 $${V}_{j}({s}_{t})={V}_{j}({s}_{t})+{\eta }_{j}[{V}_{j}({s}_{t+1})-{V}_{j}({s}_{t})]$$

where *η*_*j*_ is the learning rate of player *j*, and *s*_*t*_ represents the state at time *t*. *V*_*j*_(*s*_*t*_) denotes the initial value, which is updated along the episode. If player 1 wins or loses the game, *V*_1_(*s*_*t*_) is set to be 1 or -1, respectively. Otherwise, *V*_1_(*s*_*t*_) is set to be 0.5, and *V*_2_(*s*_*t*_) follows the same rule.

## Supplementary information

Supplementary Information

Transparent Peer Review file

## Data Availability

The reconstruction results generated in this study have been deposited in the Figshare database under the Creative Commons Attribution 4.0 International Public License and can be accessed via 10.6084/m9.figshare.31829287. This study made use of a publicly available benchmark datasets COVID-19 radiography database.

## Code availability

The codes in this study have been deposited on the Code Ocean database under the MIT License at 10.24433/CO.5665610.v1.
