# Supplementary material for: Large-scale integrated optoelectronic chaos for machine learning acceleration
Source: Nat Commun. 2026 Jun 12;17:7488. doi: 10.1038/s41467-026-73440-2 (PMC13408576; doi:10.1038/s41467-026-73440-2)
Supplement: Supplementary file 1 — Supplementary Information [file 41467_2026_73440_MOESM1_ESM.pdf]

Supplementary information for: Large-scale integrated optoelectronic chaos for machine learning acceleration

Zhouyang Pan[1†], Zhekai Zheng[1†], Ping Li[1], Hao Wang[2], Jiacheng Guo[1], Ding Cui[1], Zhihui Li[3], Jiaqi Shen[1], Lihan Wang[1], Mengya Zong[1], Simin Li[1], Zhe Kang[1], Yue Yuan[4], Jianqi Hu[5], Jijun He[1*], Yuxin Liang[3*], Dan Zhu[1*], Shilong Pan[1*]

[1*]National Key Laboratory of Microwave Photonics, Nanjing University of Aeronautics and Astronautics, Nanjing 210016, China.
[2]Department of Precision Instrument, Tsinghua University, Beijing 100084, China.
[3]United Microelectronics Center, Chongqing, China.
[4]Department of Cardiology, The First Affiliated Hospital with Nanjing Medical University, Nanjing 210029, China.
[5]Department of Electrical and Electronic Engineering, The University of Hong Kong, Hong Kong, China.

*Corresponding author(s). E-mail(s): jijun.he@nuaa.edu.cn; yuxin.liang@cumec.cn; danzhu@nuaa.edu.cn; pans@nuaa.edu.cn;
Contributing authors: pan_zy@nuaa.edu.cn; zhengzk@nuaa.edu.cn; li_ping@nuaa.edu.cn; h-wang20@mails.tsinghua.edu.cn; guojc452@nuaa.edu.cn; cdjinge@nuaa.edu.cn; zhihui.li@cumec.cn; jqshen@nuaa.edu.cn; andwwlh@nuaa.edu.cn; myzong@nuaa.edu.cn; lisimin@nuaa.edu.cn; zhe.kang@nuaa.edu.cn; yuanyue@njmu.edu.cn; jianqi@hku.hk;
†These authors contributed equally to this work.

Contents

Supplementary Notes:

1. Fundamental characteristics of the chips employed in the realized iMOCE architecture
2. MAB problem acceleration
3. Traveling salesman problem acceleration

Supplementary Figures:

Figure S1. Fundamental characteristics of the chips employed in the realized iMOCE architecture
Figure S2. Results of the MAB problem acceleration
Figure S3. Results of the traveling salesman problem acceleration

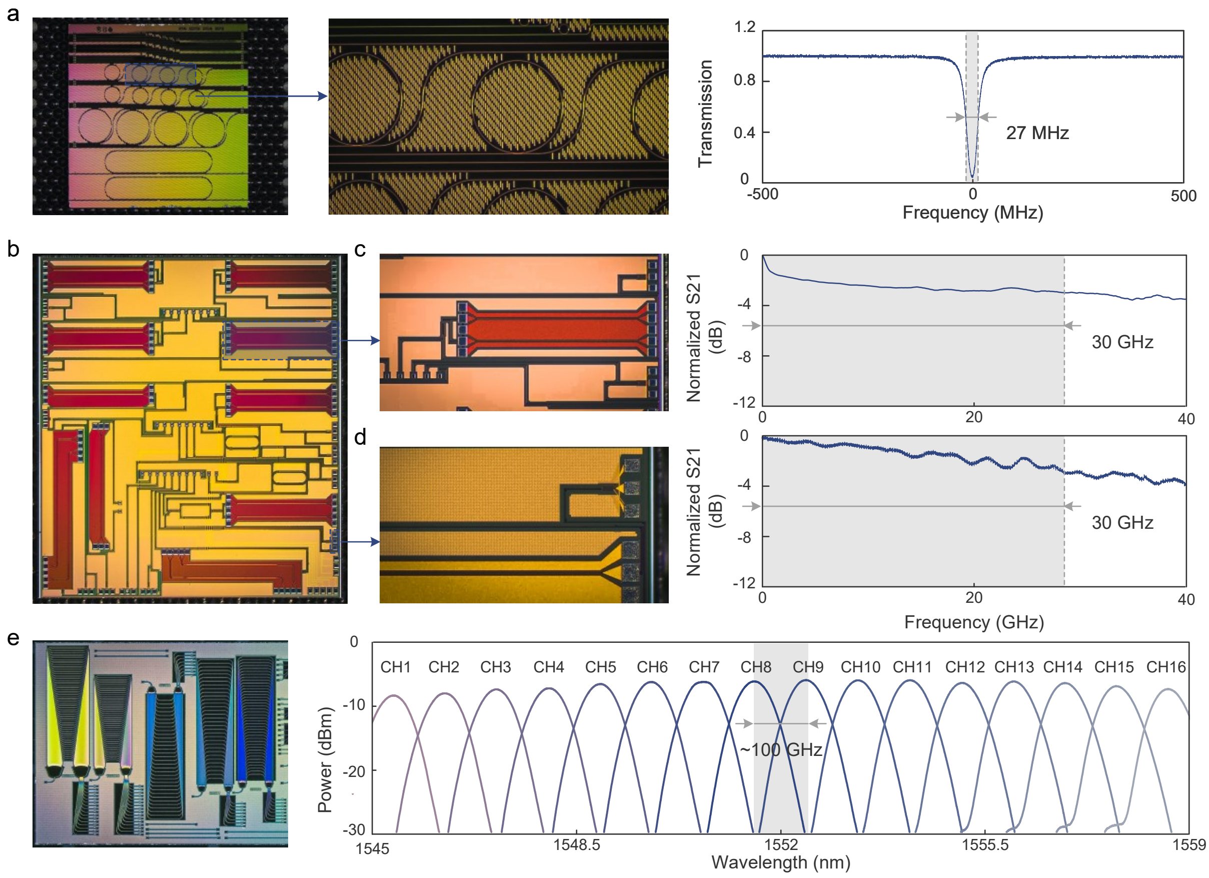

Figure S1. **a-d** Optical images and main performance of several fundamental devices, including a MRR (**a**), a MZM (c), a PD (**d**), and a WDM (**e**).

Supplementary Note 1. Fundamental characteristics of the chips employed in the realized iMOCE architecture

Figures S1a–e summarize fundamental characteristics of the chips employed in the realized iMOCE architecture. In the experiment, a continuous-wave pump at a wavelength of 1550.3 nm from a narrow-linewidth laser is amplified to 25 dBm by an EDFA. The output optical signal is coupled into a silicon-nitride MRR with a radius of 227 μm and a waveguide cross-section scale of 0.8×2 μm. The optical images and performance of the MRR are illustrated in Figure S1a, demonstrating a FWHM of 27 MHz and a Q of 7×10^6. The temperature of the MRR is stabilized at 23 ± 0.1 °C via a TEC. An optical filter selects comb lines that are routed to the OENC. The OENC closes the feedback loop by coupling an electro-optic Mach–Zehnder modulator (Figure S1c) with a 3-dB bandwidth of 30 GHz to a photodetector (Figure S1d) with a 3-dB bandwidth of 30 GHz, generating broadband chaos. The multi-channel chaotic output is demultiplexed by a WDM. The WDM receiver employs a 16-channel arrayed-waveguide grating that, upon injection of the amplified comb, selectively demultiplexes target lines and routes them to the PD array. The measured channel spacing is about 100 GHz, in good agreement with the free-spectral range of the microcomb, enabling low-crosstalk parallel operation within the target bandwidth. Notably, the MRR and WDM tested in the study are fabricated in the Chongqing United Microelectronics Center, and the OENC is fabricated in the Nanjing Nanzhi Advanced Optoelectronic Integrated Technology Research Institute,

Supplementary Note 2. MAB problem acceleration

To demonstrate the ability of the iMOCE architecture to accelerate ML problems, the generated parallel chaos is applied to an MAB problem, as shown in Figure S2. In this scenario, N slot machines each offer a reward possibility $P_i (i \in [1, N])$. The goal is to identify the slot machine with the highest P_i through iterative exploration. During the exploration process, the parallel chaotic channels are time-division multiplexed to form N sub-series, with each

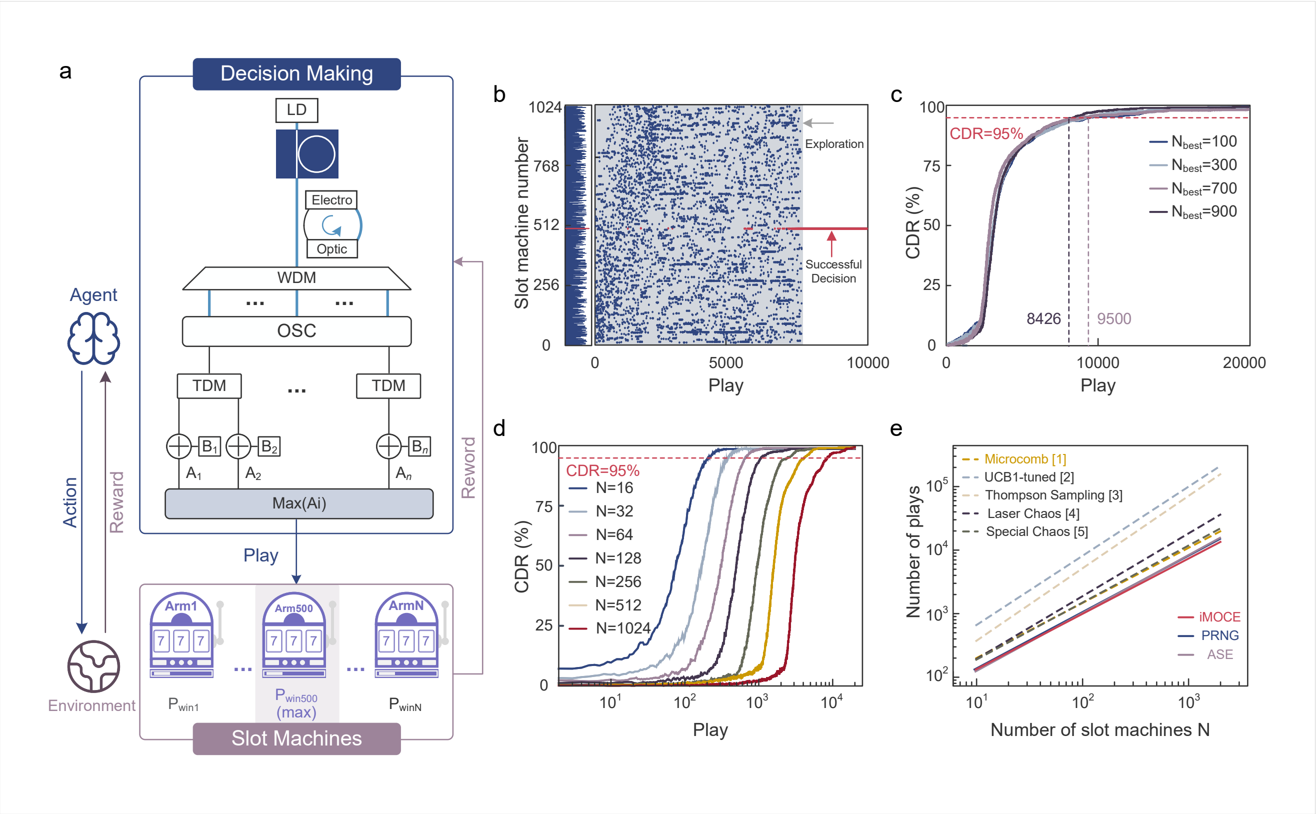

Figure S2. **a.** The setup of the MAB problem based on the iMOCE. Parallel chaotic channels are time-division multiplexed into multiple sub-series, each mapped to one arm. The generated chaos matrix is combined with the value matrix to compose the bias matrix. In each cycle, the slot machine i with the highest biased score $A_i(t)$ is played. This balances exploration and exploitation and supports efficient convergence under non-stationary rewards. **b.** The decision process of the 1024-armed MAB problem, where the left figure indicates the initial probability distribution of the slot machines. **c.** The trend of the corrected decision rate (CDR) with the increase of plays, where the red dot line marks the CDR of 95%. **d.** The trend of the CDR under different scales. **e.** The comparison between the iMOCE and other optical chaotic sources. The curves also compare the iMOCE with a digital PRNG and optical ASE noise. The overlapping results confirm that iMOCE achieves equivalent algorithmic performance to standard high-quality random sources.

sub-series representing one slot machine. In each cycle t, the slot machine i with the highest biased value $A_i(t)$ is selected and played, which can be mathematically described as

$$A_i(t) = X_i(t) + kB_i(t) \tag{1}$$

where $X_i(t)$ and $B_i(t)$ represent the chaos sequence and value estimation, respectively. k is the value coefficient, which strongly influences the balance between exploration and exploitation. A smaller k keeps the dynamics closer to unbiased random exploration, requiring more plays to select the best arm. A larger k amplifies the current bias and accelerates exploitation, but risks locking onto a sub-optimal arm. In this problem, k is set to 0.08. The value estimation $B_i(t)$ is determined based on the tug-of-war method, which is given by

$$\begin{aligned} B_i(t) &= Q_i(t) - \frac{1}{N-1}\sum_{i' \neq i}^{N} Q_{i'}(t) \\ Q_i(t) &= \Delta W_i - \gamma L_i \\ \Delta &= 2 - \gamma \\ \gamma &= \hat{P}_{\text{top1}} + \hat{P}_{\text{top2}} \\ \hat{P}_i &= \frac{W_i}{T_i} \end{aligned} \tag{2}$$

where T_i is the total time, W_i and L_i are the time of win and loss, respectively. $\hat{P}_{top1}$ and $\hat{P}_{top2}$ are the highest and second-highest estimated hit probabilities. The above equations dynamically balance exploration (via chaotic sequence $X_i(t)$) against exploitation (via value estimation $B_i(t)$), and ensure that the system efficiently converges to the optimal arm under nonstationary reward conditions.

Figure S2b presents the choice outcomes for a 1,024-armed bandit problem, where reward probabilities are set as follows: $P_1 = 0.62, P_2 = 0.30, \ldots, P_{17} = 0.79, \ldots, P_{500} = 0.9, \ldots, P_{851} = 0.80, \ldots, P_{1024} = 0.50$. For all non-optimal arms, the reward probability P_i is independently selected from 0.3 to 0.8. For the optimal arm, the reward probability P_i is set to 0.9. It's obvious that the iMOCE accelerator rapidly converges to the optimal arm. To quantify performance, 500 plays constitute one cycle, and the correct-decision rate (CDR) is defined as the fraction of plays in which the optimal arm is chosen. A cycle is deemed a converged cycle (CC) once CDR exceeds 95 %. Figure S2c tracks CDR evolution for different best-arm indices over successive cycles. Figure S2d shows the convergence cycles as a function of N, and Figure S2e compares this method with existing algorithms [1–5]. Using power function fitting, the relationship between the convergence period N_{CC} and the number of slot machines N can be described as $N_{CC} = 20.6 \cdot N^{0.86}$. For the microcomb, laser chaos, and special chaos system, it follows the same selection rules (tug-of-war algorithm) as the iMOCE system, while the chaos is generated by a microcomb, chaotic laser, or an OENC. The relationship between the convergence period and the number of slot machines is $N_{CC} = 26.48 \cdot N^{0.89}$, $N_{CC} = 22 \cdot N^{0.98}$, and $N_{CC} = 30 \cdot N^{0.86}$, respectively. For the UCB1 algorithm, it follows the standard formulation listed in reference [2]. At play t, letting $\mu_i(t)$ be the empirical mean reward of arm i and $n_i(t)$ the number of times arm i has been played, the chosen arm can be expressed as

$$i_t = \arg\max_i \left[\mu_i(t) + \sqrt{\frac{2\ln t}{n_i(t)}} \right]. \tag{3}$$

The relationship between the convergence period and the number of slot machines is $N_{CC} = 59.2 \cdot N^{1.08}$. The Thompson sampling algorithm follows the standard formulation listed in reference [3]. Rewards are modeled as Bernoulli variables. For each arm, a Beta posterior Beta(α_i, β_i) is maintained with a uniform prior $\alpha_i = \beta_i = 1$. At play t, the chosen arm can be expressed as

$$i_t = \arg\max_i \theta_i^t, \tag{4}$$

where θ_i^t is drawn from Beta(α_i, β_i). After observing the reward r_t, the posterior is updated by setting $\alpha_{i\text{-}t} + r_t$ to $\alpha_{i\text{-}t}$, and $\beta_{i\text{-}t} + 1 - r_t$ to $\beta_{i\text{-}t}$, respectively. The relationship between the convergence period and the number of slot machines is $N_{CC} = 30 \cdot N^{1.13}$. As compared favorably with published approaches, the proposed scheme demonstrates faster convergence and fewer required explorations. Consequently, the iMOCE accelerator locates the optimal arm more rapidly, substantially enhancing the overall system performance. To further distinguish the role of the random source from that of the tug-of-war update, the MAB tasks are repeated using a digital pseudo-random number generator (PRNG), and optical amplified-spontaneous-emission (ASE) noise shaped by the setup in Figure 2h. As shown in Figure S2e, the three curves almost overlap and follow a similar power-law scaling with N. This situation confirms that iMOCE provides randomness that is equivalent to high-quality PRNG and ASE noise for this task, while offering much higher physical throughput and scalability.

Supplementary Note 3. Traveling salesman problem acceleration

To evaluate the efficacy of the realized iMOCE architecture in accelerating global optimization problems, the Hopfield-network-based traveling-salesman problem is tested. The Hopfield-network accelerator encodes constrained optimization problems as an "energy" function E, whose minimization identifies optimal solutions for NP-hard problems such as the traveling-salesman problem and the max-cut problem. As shown in Figure S3b, a well-known limitation of this network is entrapment in local minima, yielding suboptimal outcomes. To solve this problem, the iMOCE architecture injects broadband chaotic fluctuations that make the solution jump out of the current local optimum, avoiding the premature convergence. Initially, a state matrix s is populated with -1 entries, where each element $s_{i,j} \in [+1, -1]$ signifies whether city i is visited at position j in the route. During each update pass of the state matrix s, the weighted feedback matrix WF of each state is calculated according to the weight matrix w and the state matrix s. The sum of the weighted feedback matrix WF and the chaos matrix is then compared with a threshold θ. The state is set as 1 when the sum value exceeds the threshold θ. Otherwise, the state is set as -1. The process iterates until all entries have been updated, yielding a final state matrix that encodes a complete tour. The network's energy E decreases monotonically as updates proceed, converging toward

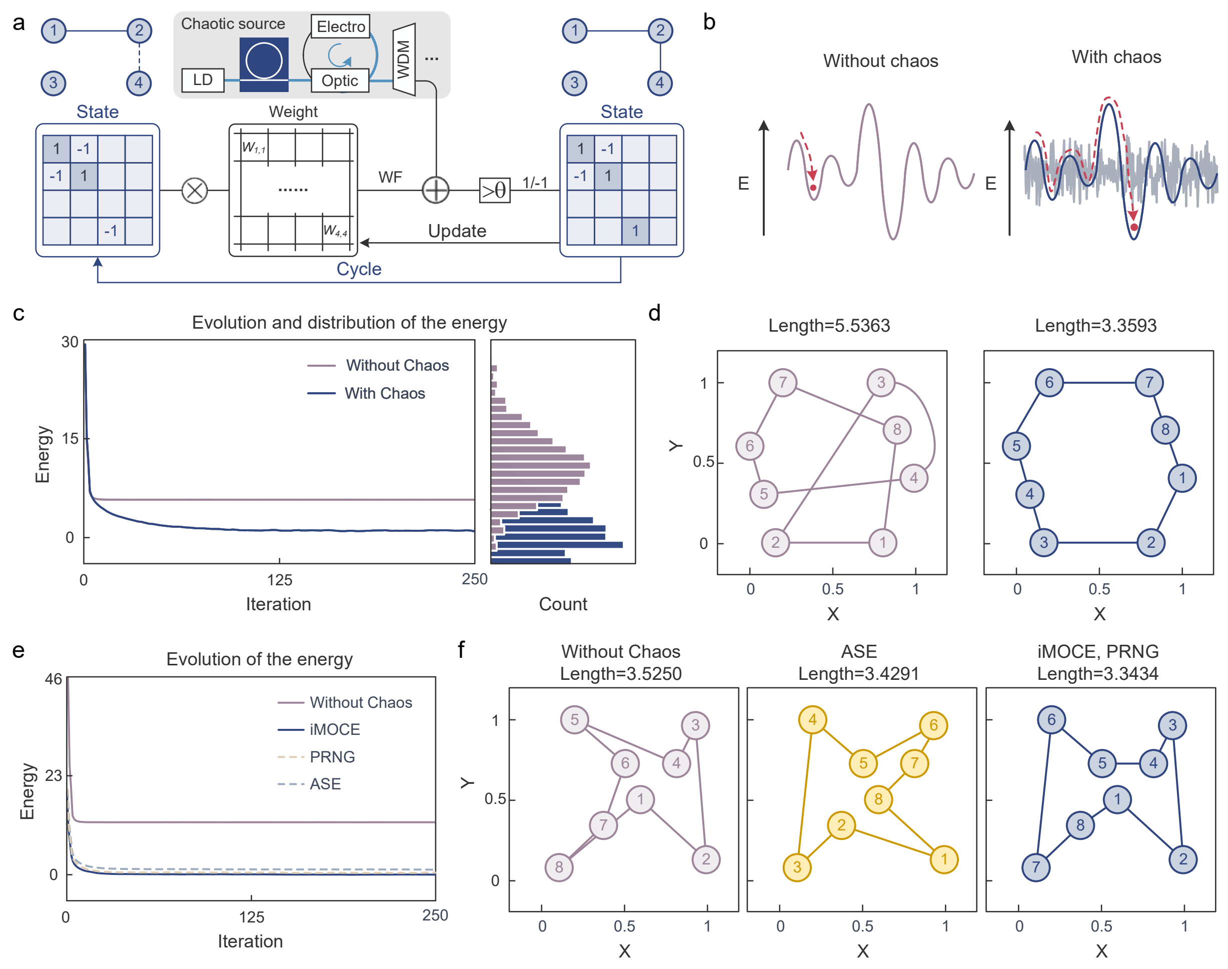

Figure S3. **a.** The setup of the traveling-salesman problem acceleration based on the iMOCE. **b.** The schematic depiction of the global minimization process enabled by chaos. Broadband perturbations from the iMOCE inject stochastic kicks that help the solver escape local minima and mitigate premature convergence. **c.** The evolution and distribution of the energy with/without chaos. Chaotic injection yields faster descent and a lower steady-state energy, with distributions concentrated near the global optimum. **d.** The results of the convergence route. With chaotic perturbations, the trajectory smooths and reaches the global optimum. Thus, the injection of physical chaos enables the system to escape local minima and prevents suboptimal trapping. **e.** The evolution of the Hopfield-network energy for a second 8-city task under four perturbation conditions, including deterministic update without chaos, and stochastic updates driven by iMOCE chaos, electronic PRNG, and optical ASE noise, respectively. **f.** The best tours found for the second 8-city task.

the nearest stable minimum, which can be mathematically described as,

$$\begin{aligned} E = {} & C_1 \sum_i \sum_k \sum_{j \neq k} s_{i,k} s_{i,j} + C_2 \sum_i \sum_k \sum_{j \neq k} s_{k,i} s_{j,i} \\ & + C_3 [\sum_i \sum_k s_{i,k} - n]^2 \\ & + C_4 \sum_k \sum_{j \neq k} \sum_i D_{k,j} s_{k,i} (s_{j,i+1} + s_{j,i-1}) \end{aligned} \tag{5}$$

where $C_1 - C_4$ represent the row-uniqueness constraint, the column-uniqueness constraint, the total-cities constraint, and the minimal-distance constraint, respectively. $D_{k,j}$ denotes the distance between city k and city j. n is the total number of cities. Thus, the tour with the minimum energy corresponds to a solution.

From this energy function, the weight matrix w is given by,

$$w_{(i,k),(l,j)} = -C_1\delta_{i,l}(1-\delta_{k,j}) - C_2\delta_{k,j}(1-\delta_{i,l}) \\ - C_3 - C_4 D_{i,l}(\delta_{j,k+1}+\delta_{j,k-1}) \tag{6}$$

where the Kronecker delta function $\delta_{i,k}$ is 1 if $i = k$ and 0 otherwise.

At each update step, the weighted feedback can be calculated as,

$$WF_{i,j} = \sum_{k,l} w_{(i,k),(l,j)} s_{k,l} \tag{7}$$

To escape local minima, a chaotic perturbation term X is added, giving the update rule,

$$s_{i,j} = \begin{cases} +1, & \text{if } WF_{i,j} + k_c X > \theta, \\ -1, & \text{otherwise,} \end{cases} \tag{8}$$

where $k_c = 1.5$ scales the influence of the chaotic fluctuation. In practice, X is supplied by the iMOCE. One optical chaos channel is photo-detected and continuously captured by a real-time oscilloscope, and the obtained samples are streamed to the Hopfield solver (one sample per neuron update step). When $k_c = 0$, this reduces to a deterministic Hopfield network that often gets trapped in local minima. When $k_c = 1.5$, the iMOCE term provides time-varying perturbations analogous to simulated annealing, enabling the network to escape minima and explore shorter tours.

In each trial, the constraint coefficients are fixed to $C_1 = 2$, $C_2 = 2$, $C_3 = 0.9$, and $C_4 = 0.21$. The threshold θ is set to 66. All 64 neurons are updated once per iteration, and the network is evolved for 250 iterations. Figure S3c indicates the trend curve of energy E over 1000 randomized instances of the traveling-salesman problem. The network without chaos rapidly settles at a local minimum, resulting in a tour length of 5.5363 m. By introducing the chaotic perturbations, the convergence evolution of the network is smoother and reaches the global optimum with a length of 3.3593 m. Thus, chaotic injection accelerates convergence and prevents entrapment in suboptimal states.

To verify that the observed effect is neither specific to one city layout nor unique to iMOCE, a second 8-city task is demonstrated. For this fixed graph, 1000 dependent trails of the Hopfield network under four conditions are tested, including without any perturbation, and with stochastic perturbations provided by iMOCE, PRNG, and ASE noise. Figure S3e plots the average energy versus iteration. Without chaos, the dynamics quickly settle into a high-energy local minimum and remain trapped, whereas all three noise sources drive the network out of the minimum and towards the global optimum. The corresponding best tours are shown in Figure S3f, where the deterministic case yields a sub-optimal route of length 3.5250, ASE noise improves this to 3.4291, and both iMOCE and PRNG perturbations converge to an identical tour length of 3.3434. These results confirm that, for the Hopfield-TSP task, iMOCE acts as a broadband physical perturbation that is algorithmically equivalent to conventional white-noise sources, while offering orders-of-magnitude higher random-bit throughput. Further tuning the chaos intensity and bandwidth may yield additional learning speed and accuracy gains.

Supplementary References

[1] Shen, B., Shu, H., Xie, W., Chen, R., Liu, Z., Ge, Z., Zhang, X., Wang, Y., Zhang, Y., Cheng, B. *et al.*, Harnessing microcomb-based parallel chaos for random number generation and optical decision making, *Nature Communications* **14**(1), 4590 (2023).

[2] Auer, P., Cesa-Bianchi, N. & Fischer, P., Finite-time analysis of the multiarmed bandit problem, *Machine Learning* **47**, 235–256 (2002).

[3] Thompson, W. R., On the likelihood that one unknown probability exceeds another in view of the evidence of two samples, *Biometrika* **25**(3/4), 285–294 (1933).

[4] Morijiri, K., Mihana, T., Kanno, K., Naruse, M. & Uchida, A., Decision making for large-scale multi-armed bandit problems using bias control of chaotic temporal waveforms in semiconductor lasers, *Scientific Reports* **12**(1), 8073 (2022).

[5] Morijiri, K., Takehana, K., Mihana, T., Kanno, K., Naruse, M. & Uchida, A., Parallel photonic accelerator for decision making using optical spatiotemporal chaos, *Optica* **10**(3), 339–348 (2023).
