# Supplementary material for: Large-scale integrated optoelectronic chaos for machine learning acceleration
Source: Nat Commun. 2026 Jun 12;17:7488. doi: 10.1038/s41467-026-73440-2 (PMC13408576; doi:10.1038/s41467-026-73440-2)
Supplement: Supplementary file 2 — Transparent Peer Review file [file 41467_2026_73440_MOESM2_ESM.pdf]

nature portfolio

Peer Review File

Large-scale integrated optoelectronic chaos for machine learning acceleration

Corresponding Author: Professor Jijun He

This file contains all reviewer reports in order by version, followed by all author rebuttals in order by version.

Version 0:

Reviewer comments:

Reviewer #1

(Remarks to the Author)
The authors combine two approaches for generating chaos---microring resonator (MRR) and optoelectronic nonlinear cavities (OENC)---to realize faster chaos bandwidth per channel in a form that can be parallelized. Moreover, the authors then integrate their scheme onto foundry silicon and silicon nitride photonic circuits and realize parallelization via wavelength division multiplexing (WDM). Overall the authors claim a chaotic bitrate an order of magnitude faster than previous approaches. Then, they use apply generated with their device to a number of machine learning demonstrations.

The text is decently written with a few small grammar issues.

I think this work is impressive from a photonic standpoint and support publication with revisions in Nature Communications. However, I have questions regarding the bigger picture and usefulness of this device, summarized in two main points:

(1) Even though this device can generate chaos at multi-terahertz rates, can this chaos be used in realtime?

It seems to me that the information pipeline remains constrained by the electronic data bandwidth of a GPU or other device. Is there an advantage to having a super-fast chaos generator if I can only upload the chaos slowly? Do I win compared to cached chaos (see next point). I think the answer to this question is that future more capable hardware will be able to take advantage of their speed, but I would appreciate some comments from the authors on this point.

(2) How does their device compare to commercial hardware for random number generation?

In the main text, the authors have in some ways setup a "strawman" (https://en.wikipedia.org/wiki/Straw_man) when arguing their point. The text compares their chaos with micro comb chaos (Fig.1-4), their chaos with "no chaos" (Fig.5b K=0, Sup.Fig.3b-d), does not compare their results at all (Fig.6), or compares with other photonic approaches (Sup.Fig.2e). However, none of these cases are what a GPU manufacturer might care about when deciding to integrate their device into a product. The "steelman" (the opposite of a strawman) is to compare with the industry standard: psuedorandom generation, other forms of hardware entropy harvesting, or cached chaos.

The authors mention in their introduction: "Digital pseudo-random and digital-chaos sources built on nonlinear analog circuits are widely used, but the throughput and processing time are constrained by the electronic bandwidth." However, based on my first point (1), it is unclear to me whether this is a major issue as cached chaos faces the same information bottleneck (uploading to GPU) as the author's approach for fast chaos generation.

If I keep a multigigabyte cache for weight initialization on my drive and also upload gigabyte cache of random numbers on my GPU for gradient descent, isn't this good enough for most machine learning optimization problems? The authors mention "For instance, next-generation mobile networks will require millisecond or sub-millisecond slice allocation and QoS management for VR/AR and vehicle-to-everything services, while autonomous vehicles and drone swarms demand collision

warnings on the order of milliseconds." Could these applications be accomplished with cached chaos? I'm not sure of the answer to these questions and I think the text would be stronger if the authors could justify their approach more. I would love to see something like Sup.Fig.2e comparing their photonic approach with cached chaos or existing industry approaches.

I have a few smaller points:

(a) "By using a micro-comb to optoelectronic nonlinear cavity..."
The "to" does not make sense here. Maybe the following is what is meant:
"By using a micro-comb [coupled to a] optoelectronic nonlinear cavity"?

(b) "The proposed iMOCE achieves a total random..."
"proposed" here implies to me that this work was theoretical (which it is not). I would suggest instead:
"The [realized] iMOCE achieves a total random..."

(c) Why is the output "intrinsically symmetric"? I might have missed it, but I don't think this was explained in the text.

(d) The link to reference 67 is bolded for some reason.

(e) Some people (section 3.2 of https://cetinkayakoc.net/docs/b08.pdf) claim that Chaos RNG is "objectionable" because measurements of physical systems might have correlations. I think Fig.4 justifies your approach, but I am curious about what you might say to such concerns. For instance, would a lower bitrate be more useful for the application most sensitive to correlations (e.g. cryptographic applications)? Perhaps the true advantage is not fast chaos generation, but rather it is better chaos generation at slow speeds compared to other chaos generators with lower bandwidth.

(Remarks on code availability)

Reviewer #2

(Remarks to the Author)
The authors present new results on an integrated setup combining microcomb generation and opto-electronic feedback for the creation of a high-bandwidth optical chaotic signal, enabling high-speed random number generation. The hardware demonstration of the random number generation is impressive and does seem to significantly exceed the previous state of the art.
There may be some merit in publication of these results.

- However, the authors should consider the energy budget of their random number generation scheme, and how it scales, especially when compared to a digital pseudo-random number generation scheme. Is there a realistic pathway to exceeding the power budget and area budget of digital computers, especially when the laser sources also needs to be integrated?

More importantly, in my view, the attempts to show "applications" and accelerations of standard tasks are raising several questions and require a more careful explanation or analysis.
Apart from the NIST test suite for RNG - which the proposed platform seems to pass well - each of the other 4 proposed tasks (2 in the main manuscript, 2 in the supplementary) is potentially flawed or requires more careful explanations.

- All of the acceleration tasks, when included, require a full list of used parameters, formulas, initialization settings etc. In principle, technical details must be given in such precision, that an external reproduction should be possible.
- It is also not clear how the random numbers of the iMOCE were used for these tasks - where the random numbers just recorded digitally and the used in a digital post-processing stage?

Open questions regarding each specific task:
The connect-3 game:
- How was the state matrix implemented? Was this all done digitally?
- It is not clear what is meant by "5000 moves over 1000 trials" - what is different between trials? The initial choice of the value matrix? What are "plays" in Fig 5 meant to be? Trials or moves?
- If there are multiple trials, there should be error bars added to Fig. 5
- For the K=0 case, does the initial choice of the value matrix strongly influence the learning? Do all different choices reach similar win rates? How was this chosen?

Heart arrythmia recognition acceleration:
- "BNN" is never spelled out in the manuscript. I assume it stands for Bayesian Neural Network, not Binary Neural Network.
- In a normal Bayesian neural network, the weights of the neurons should be sampled from some distribution. It does not look like this is the case here - instead the activations of the neurons itself seem to be modified by noise in Fig 6 a?
- It is said that, the chaotic samples are "propagated through the network weights", but the process is not mathematically well described, so it is hard to judge, but such an additive noise on activations does not obviously correspond to any Bayesian update. It would rather just reflect a regularization by noise.
- What was the exact learning rule, rates etc.? Reproducible for an external reader!

- The acceleration through the iMOCE would also only apply to this case, if the calculation of the BNN is bottlenecked by the creation of random numbers. Is this the case?
- The shown confusion matrix and data visualization is nice, but they represent a property of the BNN and learning algorithms, not of the iMOCE? Or would the authors claim that the iMOCE provided any actual performance than a different random number generator? I am explicitly not talking about efficiency. Is there any difference from white noise generated any other way?

Multi-armed bandits (from the Supplementary):
- The caption of Fig 2 a in the supplementary claims that in each cycle, the machine "with the highest biased matrix" is played. This should probably refer to the highest entry of the matrix? Otherwise the authors need to explain how an entire matrix is evaluated as being "highest".
- How the actual setting of the arms was created is important. Listing just a few entries is insufficient, instead the authors should describe the process of assigning P_i
- Figure (2 a) e shows a comparison between different MAB methods. The iMOCE is shown to solve the problems faster than other methods. This seems quite frankly a questionable claim. Don't the authors espouse the fact that the iMOCE produces very nice, symmetric chaotic signals that do not require complex post-processing? Then what special property should give these chaotic signals an edge in this task?
- Followingly, the authors should also compare if the performance is identical to pseudo-random-number generated white noise.
- The authors must give the exact settings and formulas used for the other methods listed inf Figure 2 a e - there are several hyperparameters for some of them.
- Did the authors optimize their own hyperparameters for the specific settings of P_i? If they compare their own MAB algorithm which they optimized for their specific distribution of P_i, it will naturally lead to a better performance compared to another method that was optimized for a different P_i drawing. For example, the distance between the best and second best arm, or the average win probability, etc. Figure 2 a e should not represent an unfair comparison. It seems likely that k=0.08 just happens to best match the P_i distribution shown, and not a general MAB case.
- I reiterate that it is not clear why iMOCE should have any better performance on this task - the iMOCE is supposed to supply random numbers for various applications, which it should do exactly as well as any other source.
- Once again, the technical details are lacking - were the matrices A and B actually implemented on chip? Max(A_i) was implemented how? Could the oscilloscope actually read all channels simultaneously or was this assembled digitally later etc.?

Traveling salesman problem:
- here the iMOCE acts as the noise term for an annealing problem, as far as I can tell. The method is described only very briefly, so it is difficult to understand what exactly has been done
- The caption of Figure (3 a) b that "without chaos" things get stuck in a local minimum, is not surprising, an annealing process does not work without a noise source. Once again, this is an application for noise, but it is not clear why this showcases anything in particular about the iMOCE, unless a significant part of this problem was actually hardware accelerated. It seems the iMOCE once again only supplied random numbers.
- White noise created by a pseudo-random number generator should have the same effect?
- Was only one graph tried? The one shown in the image? The setting is lacking technical details. The text mentions 1000 random initializations, but the sizes, settings etc. are not mentioned.

All these points need to be corrected.

Minor issue:
- the word 'Reward' is misspelled as "Reword" in several figures.

(Remarks on code availability)

Version 1:

Reviewer comments:

Reviewer #1

(Remarks to the Author)
As mentioned previously, I think this work is interesting and impressive from a photonics perspective and the results of Fig 4 already prove performance. However, I still share the skepticism of the other reviewer regarding the quality of the other demonstrations using their generated chaos.

While I thank the authors for taking the time to augment their demonstrations (Fig 6, S2, S3) with comparisons to other forms of randomness, I feel that the authors may have missed the point of the criticism of myself and the other reviewer. The demonstrations in Fig. 5, 6, S2, S3 are not experiments that show the compelling advantage of iMOCE over standard RNG approaches.

For instance, results that happen to show iMOCE as 'best performing' compared to PRNG and ASE (such as Fig 6i, S2e, S3e) might result from subtle differences in the shape or magnitude of the probability distribution rather than the quality of the noise in the first place. A small-scale 8-node traveling salesman problem does not seem to be the sort of problem that is highly sensitive to the sort of correlation biases that could cause issues in PRNG and ASE. These problems do not seem to be hardware accelerated to use the speed of iMOCE.

In contrast, an application showing that the speed or quality of their noise is better than PRNG would be compelling. For instance, the authors mention that PRNG/etc "can introduce correlation biases and weaken coverage of the policy space". It would be compelling if iMOCE was shown to avoid these issues for an important application, or shown to compute very fast. However, I recognize that this may be difficult or expensive to show in experiment and again find the results in Fig 4 to be more than sufficient by itself as a demonstration.

It could be argued that the demonstrations past Fig 4 (apart from MAB, which shows advantage over other photonic approaches and appears to be a standard benchmark for hardware accelerators) detract from the quality of the manuscript because they do not show strong advantage over existing techniques. I will leave it to the editor to decide if they would prefer some of the content to be trimmed. For publication in Nature Communications, I would be satisfied if the authors were to take slightly more care with their language and claims regarding Fig. 5, 6, S2, S3.

For example:

(a)

"The utility and generality of the multi-channel large-bandwidth chaotic entropy sources are validated by benchmarking five representative tasks, including the[omit: grammar] random bit generation, MAB, connect-3, traveling-salesman solving, and electrocardiogram (ECG) trace recognition. By integrating optical chaos on a wafer-scale integrated photonic platform, iMOCE functions as a reproducible computing resource that delivers end-to-end gains on heterogeneous ML workloads and is poised for system-level deployment."

'utility and generality' being 'validated' is a stretch because it seems that the authors do not use the 'large-bandwidth' or 'multi-channel' nature of their device in the demonstrations. 'delivers end-to-end gains on heterogeneous ML workloads' is a stretch, I do not think the authors do not demonstrate gains over existing techniques.

(b)

As an example of a well-posed claim, I approve of the accuracy of the end of the heart arrhythmia section:

"This confirms that the iMOCE acts as a high-quality physical random engine that can replace conventional noise sources without degrading accuracy."

Here, the authors claim exactly what they did.

(Remarks on code availability)

Reviewer #2

(Remarks to the Author)
The authors have presented an updated version of their manuscript on a new proposal for high-speed generation of random numbers via microcombs and opto-electronic feedback for on-chip integration.

The main appeal of the paper remains in the physical setup and experimental demonstrations. The authors have presented some additional arguments, but there remain some doubts on the real-world usability of such a large quantity of random numbers. Generously speaking, this reflects the fact that the RNG-side is ahead of potential other components needed for system-scale use, e.g., photonic neural networks that might profit from it. Therefore, the experimental side of the paper presents a sufficient advance to merit publication in my view.

On the demonstrations side, I think the clarifications now added by the authors were very necessary. The original manuscript suffered from a lack of details, that would leave a casual reader with wrong impressions that the iMOCE itself is responsible for solving heart arrythmia, traveling-salesman, decision making et cetera. The new comparisons to PRNG and ASE correctly show that it is merely a valid replacement for alternative RNG sources, and therefore correctly shifts the focus of the paper to the hardware aspects.
Traces of this "overselling" still remain, in my view. From a principled perspective, showing the passing of the NIST suite should be sufficient. I will admit that the expanded set of examples in the manuscript helps to illustrate how various forms of RNG can be used across different aspects of machine learning and optimization algorithms, and therefore not demand any further changes.

Overall, the paper is now suitable for publication from my perspective.

(Remarks on code availability)
I could not find any available code to review.

Version 2:

Reviewer comments:

Reviewer #1

(Remarks to the Author)
I thank the authors for their revisions and support publication.

(Remarks on code availability)
I could not access the Code Ocean repository without making an account, which I would prefer to not do.

Reviewer #2

(Remarks to the Author)
The authors have satisfied my previous comments; I see no major remaining issues.

(Remarks on code availability)
The code-ocean capsule could profit from a README file.

Open Access This Peer Review File is licensed under a Creative Commons Attribution 4.0 International License, which permits use, sharing, adaptation, distribution and reproduction in any medium or format, as long as you give appropriate credit to the original author(s) and the source, provide a link to the Creative Commons license, and indicate if changes were made.
In cases where reviewers are anonymous, credit should be given to 'Anonymous Referee' and the source.
The images or other third party material in this Peer Review File are included in the article’s Creative Commons license, unless indicated otherwise in a credit line to the material. If material is not included in the article’s Creative Commons license and your intended use is not permitted by statutory regulation or exceeds the permitted use, you will need to obtain permission directly from the copyright holder.
To view a copy of this license, visit https://creativecommons.org/licenses/by/4.0/

We are grateful that our manuscript has been reviewed by two reviewers. We appreciate that the reviewers read it carefully, offered suggestions, and provided an overall positive evaluation. We have carefully considered every issue raised by the reviewers and made the necessary revisions to improve the manuscript. In this response, we address all concerns and comments in a point-to-point manner. The reviewers' comments are in black, our responses are in blue, and the revisions made in the revised manuscript are in red.

Reviewer #1

The authors combine two approaches for generating chaos---mirroring resonator (MRR) and optoelectronic nonlinear cavities (OENC)---to realize faster chaos bandwidth per channel in a form that can be parallelized. Moreover, the authors then integrate their scheme onto foundry silicon and silicon nitride photonic circuits and realize parallelization via wavelength division multiplexing (WDM). Overall, the authors claim a chaotic bitrate an order of magnitude faster than previous approaches. Then, they apply generated by their device to several machine learning demonstrations.

The text is decently written with a few small grammar issues.

I think this work is impressive from a photonic standpoint and supports publication with revisions in Nature Communications. However, I have questions regarding the bigger picture and the usefulness of this device, summarized in two main points:

(1) Even though this device can generate chaos at multi-terahertz rates, can this chaos be used in real-time?

It seems to me that the information pipeline remains constrained by the electronic data bandwidth of a GPU or other device. Is there an advantage to having a super-fast chaos generator if I can only upload the chaos slowly? Do I win compared to cached chaos (see next point)? I think the answer to this question is that future, more capable hardware will be able to take advantage of their speed, but I would appreciate some comments from the authors on this point.

Response 1:

Thanks for the insightful comments. We agree that the electronic I/O bandwidth is a critical constraint in current hybrid systems. Regarding whether the optical chaos is usable in real time and whether it offers advantages over cached chaos, our clarifications are as follows.

(1) Uploading every chaotic sample to a GPU would indeed make the rate of I/O rather than the bandwidth of the source become the limiting factor. But the decision cycles of our RL or classification tasks are much shorter than the correlation time of the chaos. As long as the correlation time is well below the electrical sampling interval and the decision-update period, continuous samples can be treated as approximately independent. Under these conditions, the throughput of the system scales with ADC, SerDes, and interconnect progress.

Meanwhile, by combining the optical chaos source with optical or optoelectronic operators (weight kernels, nonlinearities, comparators, etc.), most random computations and decisions are performed at the front end. The GPU/CPU receives only highly compressed decisions or a small set of statistics, which significantly accelerates the speed of decision-making. Such architectures have been demonstrated in recent works on photonic tensor cores and probabilistic photonic computing [R1, R2].

To directly demonstrate that the realized iMOCE can be integrated with a photonic computing

kernel, we performed an additional proof-of-concept experiment on implementing a Bayesian neural network (BNN) for ECG classification driven by such large bandwidth and parallelism chaos. The experimental structure follows the scheme of reference [R3], where the generated nine chaotic comb lines from the iMOCE are shaped to encode the weights, followed by an MZM to map the ECG data to the optical carriers. And dispersive fiber propagation followed by photodetection performs the convolution in the optical domain. The subsequent nonlinear activation is implemented electrically. For a grid of target means and standard deviations, we programmed the waveshaper and measured the corresponding output over 100 random realizations. As shown in Figure R1b, the measured means and standard deviations of 10000 points closely follow the ideal diagonal line, with small deviations around the target values. The computation error of the mean and the standard deviation is 0.073 and 0.048, respectively. Using this front-end, we then trained a 3-layer BNN to classify nine ECG rhythm classes. The confusion matrix in Figure R1c shows that the iMOCE-driven BNN reaches a recognition accuracy of 95.3%. The mutual-information distributions in Figure R1e reveal a clear separation between correctly classified and OOD ECG rhythms, demonstrating that the iMOCE-driven BNN preserves the expected Bayesian uncertainty structure.

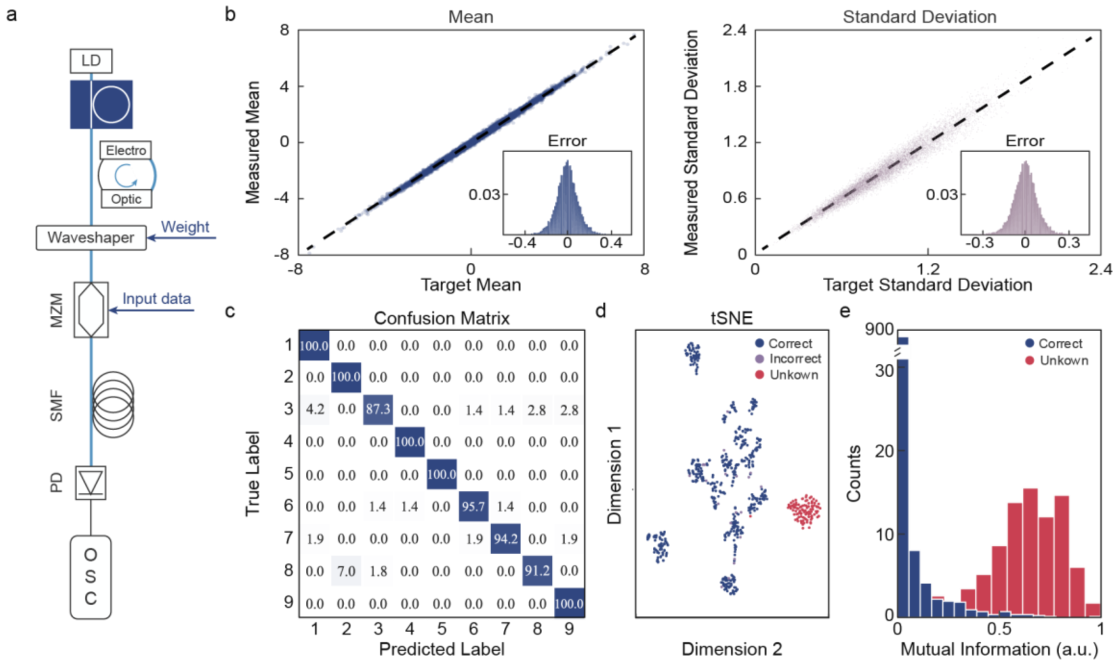

Figure R1 a. Schematic of the optical convolution setup integrating the iMOCE. The chaotic comb is spectrally shaped to encode probabilistic weights, modulated by ECG data via a Mach-Zehnder modulator (MZM), and processed through a dispersive single-mode fiber (SMF) for optical convolution before photodetection (PD). b. Accuracy verification of the photonic Bayesian machine. The scatter plots compare the measured mean (left, blue) and standard deviation (right, purple) of the output distributions against the target values. c. Confusion matrix of the ECG arrhythmia recognition task, achieving an overall accuracy of 95.3%. d. t-SNE visualization of the input ECG data embeddings. e. Mutual information distributions result. The clear separation enables effective rejection based on uncertainty.

(2) Regarding the reviewer's question on the comparison to the cached chaos, we believe that the physical real-time chaotic source used here has at least two advantages over pre-generated and stored sequences. First, it requires less storage and bandwidth overhead. For large-scale parallel workloads, each channel requires a long, non-repeating random sequence for each trial. If one relies entirely on cached chaos, each channel must be employed with a sequence whose length grows linearly with the task size and must

be streamed at high bandwidth from DRAM/HBM to the compute units. In contrast, a wideband physical chaotic source naturally provides entropy flux that scales in time and across channels without explicitly storing and transmitting very long sequences.

Second, it has the advantages of independence and unpredictability. Cached chaos will repeat or be reused after sufficient time, which introduces hidden correlation bias in tasks that require long-horizon exploration and potentially weakens the coverage of the policy space. The proposed physical real-time chaotic source provides an effectively unbounded non-repeating sequence beyond its correlation time and is physically unpredictable.

With higher-bandwidth ADCs, on-chip amplifiers, and heterogeneous optoelectronic accelerator chips in the future, more of the random sampling, energy accumulation, and nonlinear activation can be moved directly into the photonic domain, further exploiting multi-channel parallelism and the wide bandwidth of our integrated chaos source.

[R1] Brückerhoff-Plückelmann, F., Borras, H., Klein, B. et al., Probabilistic photonic computing with chaotic light, *Nature Communications* 15(1) (2024) 10445.

[R2] Choi, S., Salamin, Y., Roques-Carmes, C. et al., Photonic probabilistic machine learning using quantum vacuum noise, *Nature Communications* 15(1) (2024) 7760.

[R3] Xu, X., Tan, M., Corcoran, B. et al., 11 TOPS photonic convolutional accelerator for optical neural networks, *Nature* 589(7840) (2021) 44–51.

Revisions:

The description has been added in the third paragraph, left column of page 9, as follows,

"For instance, next-generation mobile networks will require millisecond or sub-millisecond slice allocation and QoS management for VR/AR and vehicle-to-everything services, while autonomous vehicles and drone swarms demand collision warnings on the order of milliseconds [67]. As such systems continue to evolve toward higher parallelism, shorter decision cycles, and stronger local security requirements, the proposed optical chaotic source can further improve throughput, real-time performance, energy efficiency, and physical unpredictability. Furthermore, by combining the optical chaos source with optical or optoelectronic operators, such as weight kernels, nonlinearities, and comparators, stochastic computation and decision-making can be performed primarily at the front end [4]. As such, the GPU/CPU will receive only highly compressed decisions or a small set of statistics, which significantly accelerates the speed of decision-making."

(2) How does their device compare to commercial hardware for random number generation?

In the main text, the authors have, in some ways, set up a "strawman" (https://en.wikipedia.org/wiki/Straw_man) when arguing their point. The text compares their chaos with micro comb chaos (Fig.1-4), their chaos with "no chaos" (Fig.5b K=0, Sup. Fig.3b-d), does not compare their results at all (Fig.6), or compares with other photonic approaches (Sup.Fig.2e). However, none of these cases are what a GPU manufacturer might care about when deciding to integrate their device into a product. The "steelman" (the opposite of a strawman) is to compare with the industry standard: pseudorandom generation, other forms of hardware entropy harvesting, or cached chaos.

The authors mention in their introduction: "Digital pseudo-random and digital-chaos sources built

on nonlinear analog circuits are widely used, but the throughput and processing time are constrained by the electronic bandwidth." However, based on my first point (1), it is unclear to me whether this is a major issue, as cached chaos faces the same information bottleneck (uploading to GPU) as the author's approach for fast chaos generation.

If I keep a multigigabit cache for weight initialization on my drive and also upload a gigabyte cache of random numbers on my GPU for gradient descent, isn't this good enough for most machine learning optimization problems? The authors mention "For instance, next-generation mobile networks will require millisecond or sub-millisecond slice allocation and QoS management for VR/AR and vehicle-to-everything services, while autonomous vehicles and drone swarms demand collision warnings on the order of milliseconds." Could these applications be accomplished with cached chaos? I'm not sure of the answer to these questions, and I think the text would be stronger if the authors could justify their approach more. I would love to see something like Sup.Fig.2e comparing their photonic approach with cached chaos or existing industry approaches.

Response 2:

(1) Thanks for the comment. For the GPU/ASIC manufacturer considering whether to integrate this device into a product, the more appropriate baselines are indeed the mature software-based pseudorandom number generators (PRNGs), various electronic hardware entropy sources (TRNGs), and pre-generated, cached chaos. In traditional offline-training scenarios, PRNGs or electronic TRNG already meet requirements well. Because optical chaotic sources provide high-dimensional chaotic distributions in space and frequency, they may offer advantages in acceleration and robustness, but they are not indispensable. Accordingly, in the revised manuscript, we have softened statements about such scenarios, emphasized the complementary roles of electronic and optical entropy sources, and added experimental comparisons against cached software chaos and white-noise chaos generated by an ASE source (see revised Figure 6, Figure S2, and Figure S3).

(2) In contrast, this work focuses on large-scale RL and probabilistic inference tasks that continually consume large amounts of physical entropy, require massive parallel random streams, or perform probabilistic computing directly at the optical front end. For example, if there are 10^4 parallel agents and each agent consumes 10^3 random samples per millisecond, the system requires 3.6×10^{13} samples per hour. Even at an 8-bit representation, caching one hour of random numbers would require on the order of hundreds of terabytes. In practice, sequences would have to be reused or replayed frequently, which can introduce correlation biases and weaken coverage of the policy space. Meanwhile, typical TRNGs deliver single-channel throughputs in the range of Mb/s to Gb/s, which is hard to support the scale of many independent random streams mentioned above. In contrast, our iMOCE achieves physical entropy of Tb/s level via multi-comb-line parallelism and broadband chaotic oscillation, and can naturally support a large number of mutually decorrelated random channels within a single photonic engine. In addition, if further combined with optical convolution or other optical-computing architecture, chaos-driven probabilistic sampling and energy accumulation can be carried out directly in the optical front end. In this way, the GPU/CPU receives highly compressed decision variables or statistics rather than a full random bitstream, thereby avoiding the "random-number upload bandwidth bottleneck" noted by the reviewer (see Figure R1).

Finally, regarding the examples in the "Decision part" (next-generation mobile networks, VR/AR, V2X, and drone swarms), we have adopted more cautious wording in the revision. We acknowledge that,

at current scales and architectures, well-designed PRNGs and caching strategies can indeed meet millisecond or sub-millisecond scheduling and QoS requirements. Our view is that as such systems continue to evolve toward higher parallelism, shorter decision cycles, and stronger local security requirements, the proposed optical chaotic source can provide further improvements in throughput, real-time performance, energy efficiency, and physical unpredictability.

Revisions:

(1) The description of the future application has been added as detailed in our Response 1.

(2) The description has been added in Figure 6 and the third paragraph, right column of page 8, as follows.

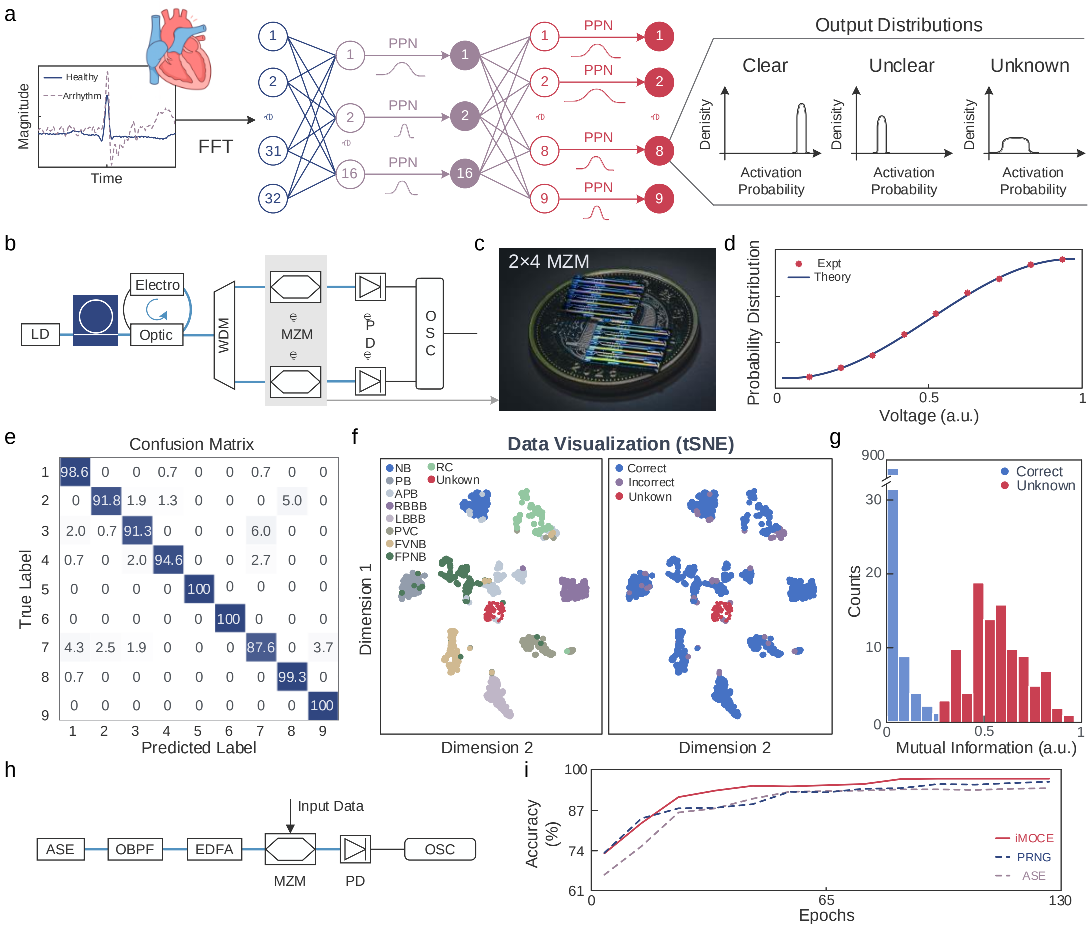

Figure 6 a. The setup of the heart arrhythmia recognition task acceleration based on the iMOCE. Each ECG trace is converted into a 32-point vector through a fast Fourier Transform (FFT) and fed to a two-layer probabilistic neural network. b. The schematic diagram of the photonics probability neuron (PPN). iMOCE supplies statistically independent chaotic samples through the network weights to realize variational posterior sampling. After training, the network outputs a posterior class-probability vector per input, from which the predicted label and epistemic uncertainty are obtained. c. The microscope photo of the MZM array. d. The probability distribution of the generated chaos as the applied voltage is changed. e. The confusion matrix of the recognition model with a recognition accuracy of 95.7\%. f. The t-SNE visualization of the input data, where nearby points correspond to similar data and distant points to dissimilar data. g. The mutual information distribution of the correct and unseen data. Mutual-information distributions for correct versus unseen data show clear separation, demonstrating successful rejection of out-of-distribution inputs. h. Experimental setup of the amplified spontaneous emission (ASE) noise-based optical noise source. i. Validation accuracy as a

function of training epochs when driven by three different random sources.

Finally, the mutual-information map in Figure 6g reveals a pronounced separation between correct and unknown samples, demonstrating successful rejection of OOD inputs.

To test the performance of the ECG task under different random sources, the probability training is repeated while the iMOCE chaos is replaced by either a digital pseudo-random number generator (PRNG) or an amplified spontaneous emission (ASE) noise-based optical white-noise source (Figure 6h). As shown in Figure 6i, all three random sources produce nearly identical learning curves and converge to final accuracy of 95.5%, 94.6%, and 92.3%, respectively. This confirms that the iMOCE acts as a high-quality physical random engine that can replace conventional noise sources without degrading accuracy.

(3) The description has been added in Figure S2 and the third paragraph of supplementary page 4, as follows.

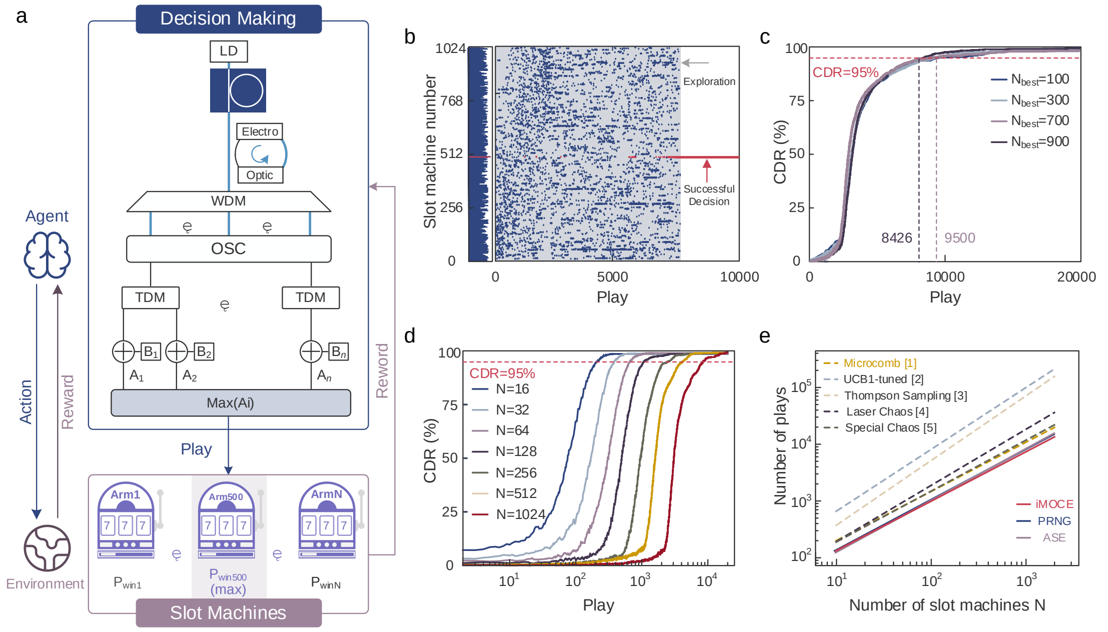

Figure S2 a. The setup of the MAB problem based on the iMOCE. Parallel chaotic channels are time-division multiplexed into multiple sub-series, each mapped to one arm. The generated chaos matrix is combined with the value matrix to compose the bias matrix. In each cycle, the slot machine i with the highest biased score $A_i(t)$ is played. This balances exploration and exploitation and supports efficient convergence under non-stationary rewards. b. The decision process of the 1024-armed MAB problem, where the left figure indicates the initial probability distribution of the slot machines. c. The trend of the corrected decision rate (CDR) with the increase of plays, where the red dot line marks the CDR of 95%. d. The trend of the CDR under different scales. e. The comparison between the iMOCE and other optical chaotic sources. The iMOCE accelerator achieves faster convergence with fewer explorations, locating the optimal arm more rapidly and improving overall performance.

Consequently, the iMOCE accelerator locates the optimal arm more rapidly, substantially enhancing the overall system performance. To further distinguish the role of the random source from that of the tug-of-war update, the MAB tasks are repeated using a digital pseudo-random number generator (PRNG), and optical amplified-spontaneous-emission (ASE) noise shaped by the setup in Figure 2h. As shown in Figure S2e, the three curves almost overlap and follow a similar power-law scaling with N. This situation confirms that iMOCE provides randomness that is equivalent to high-quality PRNG and ASE noise for this task, while offering much higher physical throughput and scalability.

(4) The description has been added in Figure S3 and the fourth paragraph of supplementary page 6, as

follows.

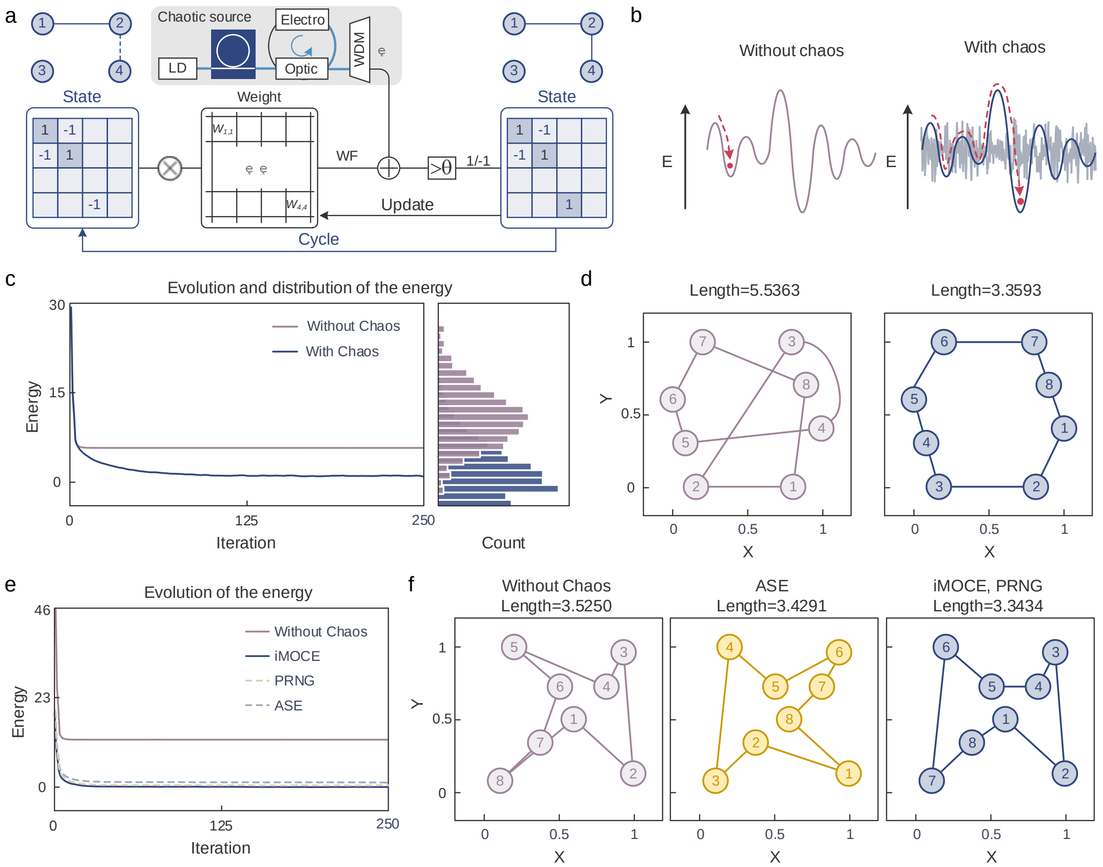

Figure S3 a. The setup of the traveling-salesman problem acceleration based on the iMOCE. b. The schematic depiction of the global minimization process enabled by chaos. Broadband perturbations from the iMOCE inject stochastic kicks that help the solver escape local minima and mitigate premature convergence. c. The evolution and distribution of the energy with/without chaos. Chaotic injection yields faster descent and a lower steady-state energy, with distributions concentrated near the global optimum. d. The results of the convergence route. With chaotic perturbations, the trajectory smooths and reaches the global optimum. Thus, chaos accelerates convergence and prevents suboptimal trapping. e. The evolution of the Hopfield-network energy for a second 8-city task under four perturbation conditions, including deterministic update without chaos, and stochastic updates driven by iMOCE chaos, electronic PRNG, and optical ASE noise, respectively. f. The best tours found for the second 8-city task.

In each trial, the constraint coefficients are fixed to C_1=2, C_2=2, C_3=0.9, and C_4=0.21. The threshold θ is set to 66. All 64 neurons are updated once per iteration, and the network is evolved for 250 iterations. Figure S3c indicates the trend curve of energy E over 1000 randomized instances of the traveling-salesman problem.

……

To verify that the observed effect is neither specific to one city layout nor unique to iMOCE, a second 8-city task is demonstrated. For this fixed graph, 1000 dependent trials of the Hopfield network under four conditions are tested, including without any perturbation, and with stochastic perturbations provided by iMOCE, PRNG, and ASE noise. Figure S3e plots the average energy versus iteration. Without chaos, the dynamics quickly settle into a high-energy local minimum and remain trapped, whereas all three noise sources drive the network out of the minimum and towards the global optimum. The corresponding best tours are shown in Figure S3f, where the deterministic case yields a sub-optimal

route of length 3.5250, ASE noise improves this to 3.4291, and both iMOCE and PRNG perturbations converge to an identical tour length of 3.3434. These results confirm that, for the Hopfield-TSP task, iMOCE acts as a broadband physical perturbation that is algorithmically equivalent to conventional white-noise sources, while offering orders-of-magnitude higher random-bit throughput. Further tuning the chaos intensity and bandwidth may yield additional learning speed and accuracy gains."

(3) "By using a micro-comb to optoelectronic nonlinear cavity..."

The "to" does not make sense here. Maybe the following is what is meant:

"By using a micro-comb [coupled to a] optoelectronic nonlinear cavity"?

Response 3:

Thanks for the suggestion. The description has been replaced in the revision manuscript.

Revisions:

The description has been added in the first paragraph, left column of page 1, as follows,

"By driving the optoelectronic nonlinear cavity with a chaotic microcomb, massively parallel channels with a 6-dB bandwidth of 25 GHz per channel are achieved."

(4) "The proposed iMOCE achieves a total random..." "Proposed" here implies to me that this work was theoretical (which it is not).

I would suggest instead: "The [realized] iMOCE achieves a total random...

Revisions:

We have made the change as suggested by the reviewer: "The realized iMOCE achieves a total random-bit generation rate of 32.768 Tbps (1.024 Tbps per channel), unparalleled by existing optical chaotic sources."

(5) Why is the output intrinsically symmetric"? I might have missed it, but I don't think this was explained in the text.

Response 5:

We thank the reviewer for pointing this out. In the original manuscript, we mainly used histogram comparisons to show that the amplitude distribution of the microcomb output is asymmetric, whereas the output of the iMOCE is approximately symmetric, but we did not clearly explain the physical reason for this symmetry.

For chaotic microcombs, modulation instability together with extreme events often cause the intensity fluctuations of individual comb lines to exhibit pronounced skewness and heavy tails, as systematically reported in prior work on random-number generation and parallel chaos [R4].

After being injected into the OENC, the photodetector converts the unipolar optical intensity fluctuations into an electrical current. The subsequent bandpass amplification chain effectively removes the DC component [R5]. As a result, the histogram of the output signal becomes statistically symmetric

centered around zero, without requiring any additional digital post-processing.

[R4] Li, P., Li, Q., Tang, W. et al., Scalable parallel ultrafast optical random bit generation based on a single chaotic microcomb, *Light Sci. Appl.* 13, 66 (2024).

[R5] Xu, Z., Tian, H., Zeng, Z. et al., Harnessing a nonlinear optoelectronic oscillator for speeding up reinforcement learning, *PhotoniX* 6, 5 (2025).

Revisions:

The description has been added in the second paragraph, right column of page 4, as follows,

"After being injected into the OENC, the photodetector converts the unipolar optical intensity fluctuations into an electrical current. The subsequent bandpass amplification chain effectively removes the DC component. As a result, the output of the iMOCE is inherently symmetric. This bypasses the complex post-processing steps, making it highly favorable for direct application in ML acceleration, as shown in Figure 3a."

(6) The link to reference 67 is bolded for some reason.

Revisions: The reference 67 has been changed back to regular.

(7) Some people (section 3.2 of https://cetinkayakoc.net/docs/b08.pdf) claim that Chaos RNG is 'objectionable' because measurements of physical systems might have correlations. I think Fig.4 justifies your approach, but I am curious about what you might say to such concerns. For instance, would a lower bitrate be more useful for the application most sensitive to correlations (e.g., cryptographic applications)? Perhaps the true advantage is not fast chaos generation, but rather it is better chaos generation at slow speeds compared to other chaos generators with lower bandwidth.

Response 7:

We thank the reviewer for raising this interesting question. In our view, the target of the critique in [Stipčević & Koç, RNG Book, Sec. 3.2] is mainly the practice of using purely deterministic chaos as a true random source. The authors claim that once the initial conditions and evolution equations are fixed, such systems provide only limited algorithmic information, which is indeed objectionable in a cryptographic sense. By contrast, our architecture treats a noise-driven chaotic system as a high-bandwidth physical entropy source and then evaluates the entropy rate and applies post-processing to control residual correlations. This is not at odds with the book's emphasis that one must handle entropy and correlation explicitly, rather than assuming chaos is inherently truly random.

In our implementation, we first whiten and symmetrize the microcomb chaos through the OENC, suppressing time-delay features and long-range memory so that the analog-domain autocorrelation approaches the noise floor after one to two delays. Meanwhile, we apply down-sampling and a bit-extraction strategy to keep the effective bit rate within a robust range. We evaluate the resulting bitstreams with the full NIST SP 800-22 test suite. As shown in Fig. 4, at the present sampling rate and extraction policy, inter-bit correlation has fallen to the level of statistical fluctuation. For RL and probabilistic inference tasks, the impact of residual correlation on final performance is negligible.

For the cryptographic setting raised by the reviewer, we agree that a more conservative extraction

configuration is required to obtain stronger guarantees and higher min-entropy bounds. With the realized iMOCE system, one can further lower the sampling rate, reduce the number of bits extracted per sample, and adopt stronger post-processing to trade throughput for larger entropy margins and lower correlation. Because iMOCE has Tb/s-class physical bandwidth, we do not face the extreme rate versus quality trade-off of low-bandwidth chaotic sources. In a sense, this matches the reviewer's point that the more meaningful advantage lies not in chasing the physical limit rate, but in delivering higher randomness quality at a given output rate.

At the same time, for the tasks we focus on in this paper (RL and probabilistic computing), we care that the random drive cannot be learned or effectively predicted at the algorithm's decision time scale. In such applications, iMOCE can simultaneously supply approximately independent random drives to multiple optical channels and a large number of agents. Notably, most of the randomness and accumulating energy can be computed at the optical front end, thereby significantly improving exploration efficiency. For these tasks, high speed and multi-channel operation are concrete advantages.

Revisions:

The description has been added in the second paragraph, left column of page 9, as follows,

"Table 2 compares these results with leading optical accelerators, highlighting the superior performance of the realized iMOCE architecture. Notably, the iMOCE can be further employed in cryptography where a more conservative extraction configuration is required to obtain stronger guarantees and higher min-entropy bounds. Because iMOCE has Tb/s-class physical bandwidth, it can further lower the sampling rate, reduce the number of bits extracted per sample, and adopt stronger post-processing to trade throughput for larger entropy margins and lower correlation."

Reviewer #2

The authors present new results on an integrated setup combining microcomb generation and opto-electronic feedback for the creation of a high-bandwidth optical chaotic signal, enabling high-speed random number generation. The hardware demonstration of the random number generation is impressive and does seem to significantly exceed the previous state of the art. There may be some merit in the publication of these results.

(1) However, the authors should consider the energy budget of their random number generation scheme and how it scales, especially when compared to a digital pseudo-random number generation scheme. Is there a realistic pathway to exceeding the power budget and area budget of digital computers, especially when the laser sources also need to be integrated?

Response 1:

We thank the reviewer for this critical question regarding the energy budget and integration pathway.

A representative digital TRNG is the SAR-ADC-based TRNG implemented in 65-nm CMOS. It delivers a rate of 1.25 Mbps and an energy per bit of 0.22 pJ/bit, and passes the NIST SP 800-22 suite [R6]. Linearly replicating such macros to reach a rate of 30 Tbps would require 2.4×10^7 independent entropy macros, with a total power consumption of 6.6 W and a total area of 2.16×10^6 mm^2. Moreover, when hundreds to thousands of mutually independent entropy streams are needed, on-chip interconnect and clock networks scale in energy and complexity.

For the present iMOCE architecture, we explicitly account for the power consumption of each key module. The measured powers are 20 mW for the external cavity diode laser module, 240 mW for the EDFA, 1.3 W for the electrical amplifier, and 1 mW for the integrated PD. With the demonstrated multi-channel entropy throughput, this corresponds to a power of 1.561 W and an energy per bit of 0.047 pJ/bit, which is comparable to or lower than the CMOS baseline even with the current bulky amplification.

Regarding the reviewer's concern about laser integration, there is a clear roadmap toward fully integrated, low-power implementations. First, the heterogeneous integration between III-V/Si lasers with ultralow-loss SiN MRR on a silicon platform has been demonstrated for on-chip soliton microcombs [R7-R8]. The self-injection locking of a DFB laser to a nonlinear MRR has been shown to enable stable and chip-scale soliton microcomb operation. In such heterogeneous integration, fiber-to-chip coupling losses are significantly reduced, and a bulky EDFA is often not required. When optical gain is needed, chip-scale erbium-doped waveguide amplifiers (EDWA) with a maximum gain of 30 dB have been demonstrated, providing an integrated alternative primarily to reduce system volume and coupling loss, while also improving end-to-end efficiency [R9]. Notably, with the above integration technology, the overall architecture is expected to be integrated in a compact module. A realistic near-term form factor is a standard 14-pin butterfly-style package with a typical footprint of about 37 mm × 13 mm, enabling a practical and scalable pathway toward system-level integration.

[R6] Jayaraj, A., Gujarathi, N.N., Venkatesh, I. and Sanyal, A., 0.6 – 1.2 V, 0.22 pJ/bit True Random Number Generator Based on SAR ADC, *IEEE Transactions on Circuits and Systems—II: Express Briefs* 67(10) (2020) 1765-1769.

[R7] Xiang, C., Liu, J., Guo, J. et al., Laser soliton microcombs heterogeneously integrated on silicon, *Science* 373(6550) (2021) 99-103.

[R8] Shen, B., Chang, L., Liu, J. et al., Integrated turnkey soliton microcombs, *Nature* 582(7812) (2020) 365-369.

[R9] Liu, Y., Qiu, Z., Ji, X. et al., A photonic integrated circuit-based erbium-doped amplifier, *Science* 376(6599) (2022) 1309–1313.

Revisions:

The description has been added in the third paragraph, left column of page 9, as follows,

"In addition, a notable strength of this approach is integration. The heterogeneous integration between III-V/Si lasers with ultralow-loss SiN MRR on a silicon platform has been demonstrated for on-chip soliton microcombs. The self-injection locking of a DFB laser to a nonlinear MRR has been shown to enable stable and chip-scale soliton microcomb operation. In such heterogeneous integration, fiber-to-chip coupling losses are significantly reduced, and a bulky EDFA is often not required. When optical gain is still required, the on-chip semiconductor optical amplifier (SOA) or an erbium-doped waveguide amplifier (EDWA) can be introduced, which primarily reduces system volume and packaging complexity while improving end-to-end efficiency. The resulting photonic accelerator can interface directly with dedicated electronic integrated circuits."

(2) More importantly, in my view, the attempts to show 'applications' and accelerations of standard tasks are raising several questions and require a more careful explanation or analysis. Apart from the NIST test suite for RNG, which the proposed platform seems to pass well, each of the other 4 proposed tasks (2 in the main manuscript, 2 in the supplementary) is potentially flawed or requires more careful explanations.
- All of the acceleration tasks, when included, require a full list of used parameters, formulas, initialization settings, etc. In principle, technical details must be given in such precision that an external reproduction should be possible.

Response 2:

Thanks for the suggestion. In the revised manuscript, we have comprehensively updated the Main Text and Supplementary Information to include all the requested parameters, formulas, and initialization settings.

For the ECG recognition task, the network architecture, optimizer settings, learning rates, and batch sizes have been detailed in the Main Text (Page 8). For the MAB task, the exact selection rules, hyperparameter *k*, and the formulas for the comparative algorithms have been added to Supplementary Note 2. For the traveling salesman problem, the Hopfield network update rules, constraint coefficients, threshold, and initialization details have been documented in Supplementary Note 3. Detailed descriptions of these revisions are provided in our specific Responses 4-22 below.

(3) It is also not clear how the random numbers of the iMOCE were used for these tasks - where the random numbers were just recorded digitally and used in a digital post-processing stage?

Response 3:

Thanks for the comment. For these tasks, the iMOCE outputs an analog RF chaotic signal that is detected by a high-speed photodetector, electrically amplified, and continuously digitized by a real-time oscilloscope with a sampling rate of 128 GSa/s. The oscilloscope streams samples to a host PC via a

gigabit Ethernet interface, where a Python pipeline performs online processing.

For the random-bit generation task, the sampled waveforms are processed in blocks, with normalization and quantization, to produce bitstreams that meet NIST SP 800-22. For the multi-armed bandit, board-game, and optimization tasks, iMOCE-generated numbers are used directly as the physical entropy source for policy sampling, thereby driving action selection or annealing perturbations. For the ECG recognition task with a Bayesian neural network, iMOCE randomness is injected into the outputs of the last layer. Aggregating multiple chaos-driven runs yields the posterior class-probability vector and associated recognition uncertainty, from which the final classification is determined.

Revisions:

The description has been added in the first paragraph, left column of page 7, as follows,

"The decision-making process is illustrated in Figure 5a. In this process, chaos generated by the 16 channels of the iMOCE architecture is transmitted to a host PC via a gigabit Ethernet interface, where a Python pipeline performs online processing."

(4) The connect-3 game:

How was the state matrix implemented? Was this all done digitally?

Response 4:

Yes, the state matrix implementation is performed entirely digitally. Specifically, the board is a discrete and digital 4×4 grid. At each time step, we flatten the board into a state vector with a length of 16, where each cell is encoded as empty, Player 1, or Player 2. Board updates, win detection, and the time-ordered log of visited states are implemented purely in software on a PC (Python). The iMOCE serves as an external physical entropy source. Its continuous chaotic waveform is sampled by the oscilloscope and streamed over Ethernet to the PC. At every decision step, we extract random numbers from this stream to sample the policy over actions.

Revisions:

The description has been added in the first paragraph, left column of page 8, as follows,

"After each game, the outcome updates the value matrix via the 1st temporal-difference learning method. Notably, board updates, win detection, and the time-ordered log of visited states are all implemented digitally."

(5) The connect-3 game:

It is not clear what is meant by "5000 moves over 1000 trials" - what is different between trials? The initial choice of the value matrix? What are the "plays" in Fig. 5 meant to be? Trials or moves?

Response 5:

In the connect-3 task, a "play" or a "move" denotes one complete game from an empty 4×4 board to a terminal outcome. And a "trial" denotes one full training run, which is composed of 5000 plays. In each trial, we initialize the value function equal to 0 and let two players fight for 5000 plays. After each play, we update the value function within the same trial. Then we independently repeat this process for

1000 trials. All trials use the same algorithm and hyperparameters. But at each decision step, the policy samples data from a distinct time segment of the iMOCE stream, yielding different exploration results.

In Fig.5, the horizontal axis "plays" is the play index k from 1 to 5000, and the vertical axis reports the average win rate at play k across the 1000 trials. To avoid ambiguity, we have revised the manuscript.

Revisions:

The description has been added to the second paragraph, left column of page 8, as follows,

"To further demonstrate the role of chaos in accelerating the game and optimizing convergence, player 1 received chaotic bias in addition to a greedy policy, while player 2 used only the greedy policy. Figures 5b-c trace the winning rates and decision-making outcomes over 1,000 independent trials, with each trial consisting of 5,000 sequential plays. The shaded regions indicate the standard error of the mean (SEM) across 1000 independent trials. In each trial, the value function is initialized as 0, and the value matrix is updated after each play. While the algorithm and hyperparameters remain constant across all 1,000 trials, the policy in each trial samples data from a distinct time segment of the iMOCE stream, yielding unique exploration trajectories."

(6) The connect-3 game:

If there are multiple trials, there should be error bars added to Fig. 5.

Response 6:

Thanks for the suggestion. In Fig.5, the horizontal axis "plays" is the play index k from 1 to 5000, and the vertical axis reports the average win rate at play k across the 1000 trials. Concretely, let $w_t(k) \in \{0,1\}$ denote whether player 1 wins the kth game in trial t. The plotted win rate is given as

$$p_t = \frac{1}{T}\sum_{t=1}^{T} w_t(k), T = 1000. \qquad (1)$$

The corresponding standard error (SEM) of the mean can be expressed as

$$SE_t = \sqrt{\frac{p_t(1-p_t)}{T}}. \quad (2)$$

The error bars have been added to the revised manuscript.

Revisions:

The error bars have been added to Fig. 5, as seen below:

"

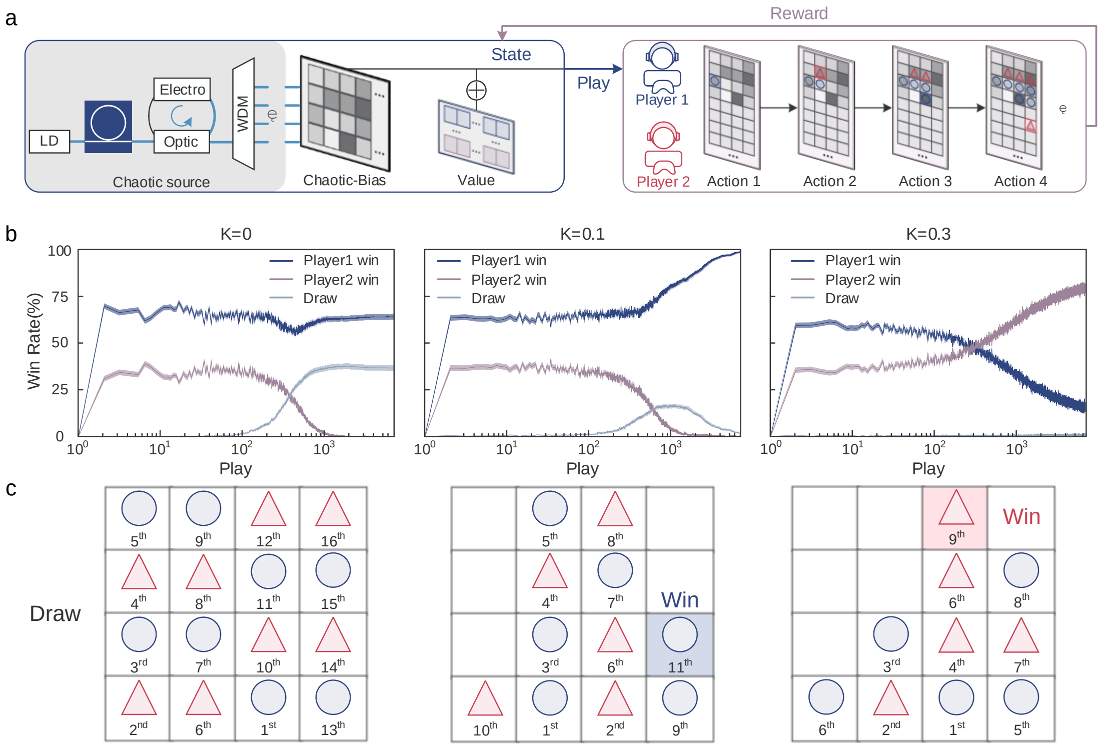

”

(7) The connect-3 game:

For the K=0 case, does the initial choice of the value matrix strongly influence the learning? Do all different choices reach similar win rates? How was this chosen?

Response 7:

Thanks for the valuable question.

For the case when K=0 (no physical chaos), at the start of each independent training trial, we set the value matrix of every board state to 0. During self-play, the value state evolves solely via TD (0) backward updates.

The initialization influences the convergence speed, but it does not overcome the fundamental limitation of the greedy policy. To explicitly address the reviewer's comment regarding the influence of initialization, we performed additional experiments modifying the initial value matrix. This corresponds to encoding a heuristic prior for the RL task [R10]. We tested two heuristic initial patterns where a fixed bias vector is added to the four columns of the board for Player 1, including a center-favoring prior [0 1 1 0] and an edge-favoring prior [1 0 0 1], respectively. As shown in Figure R2, the center-favoring prior [0 1 1 0] leads to a slightly faster increase of Player 1's win rate in the early phase, while the edge-favoring prior [1 0 0 1] slows down learning. This behavior is consistent with the combinatorial structure of the connect-3 game. The two central columns have more potential winning lines (horizontal, vertical, and diagonal three-in-a-row configurations) than the edge columns, and therefore offer a larger number of winning opportunities. However, it is worth noting that while informative priors affect the early learning speed, the final win rates in the absence of chaos (K=0) remain at suboptimal levels compared

to the chaos-driven approach, confirming that initialization alone cannot fully substitute for the exploration capability provided by the iMOCE.

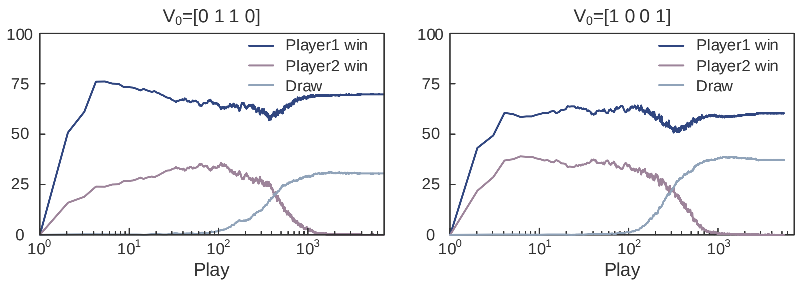

Figure R2 The win rate under different heuristic initial patterns

[R10] Sutton, R. S. & Barto, A. G. Reinforcement Learning: An Introduction. (*MIT Press*, Cambridge, MA, 1998).

Revisions:

The description of the experimental setting has been added as detailed in our Response 5.

(8) Heart arrhythmia recognition acceleration:

"BNN" is never spelled out in the manuscript. I assume it stands for Bayesian Neural Network, not Binary Neural Network.

Response 8:

Thanks to the reviewer for pointing this out. Yes, "BNN" refers to the Bayesian Neural Network.

Revisions:

The description has been added in the third paragraph, left column of page 8, as follows,

"To evaluate the ability of the iMOCE architecture in accelerating the class recognition task, a probabilistic neural network is constructed to discriminate nine clinically relevant arrhythmia categories from ECG traces."

(9) Heart arrhythmia recognition acceleration:

In a normal Bayesian neural network, the weights of the neurons should be sampled from some distribution. It does not look like this is the case here - instead, the activations of the neurons themselves seem to be modified by noise in Fig. 6a?

Response 9:

Following the reference [R2], we build a chaos-driven probabilistic neural network. All weights are pretrained as point estimates using the standard training method. The physical randomness is injected into the layer nodes, and uncertainties are obtained by aggregating multiple stochastic forward passes.

In addition, the physical randomness is injected into the weight of the network in the newly added experiment, as shown in Figure R1 (To reviewer 1).

[R2] Choi, S., Salamin, Y., Roques-Carmes, C. et al., Photonic probabilistic machine learning using quantum vacuum noise, *Nat. Commun.* 15, 7760 (2024).

Revisions:

The description of the probabilistic neural network has been added as detailed in our Response 9.

(10) Heart arrhythmia recognition acceleration:

It is said that the chaotic samples are "propagated through the network weights", but the process is not mathematically well described, so it is hard to judge, such an additive noise on activations does not obviously correspond to any Bayesian update. It would rather just reflect a regularization by noise?

Response 10:

The reviewer is correct that our implementation differs from a strict BNN where weights are sampled from a posterior distribution. As detailed in the revised manuscript, the weights in our main experiment are trained as point estimates, while the chaotic samples from the iMOCE provide noise injected directly at the layer activations. Mathematically, repeating the forward pass for the same input with this injected noise yields Monte Carlo samples of a random function. From these samples, we estimate the predictive distribution and its uncertainty. This approach aligns with the Bayesian-style noise-injection approximation methods [R2]. It differs from standard "regularization by noise" because the randomness is retained and actively sampled during inference time, rather than being used solely during training. This allows the randomness to construct the predictive distribution and quantify epistemic uncertainty.

[R2] Choi, S., Salamin, Y., Roques-Carmes, C. et al., Photonic probabilistic machine learning using quantum vacuum noise, *Nat. Commun.* 15, 7760 (2024).

Revisions:

The description has been added in the third paragraph, left column of page 8, as follows,

"Notably, the weights are trained as point estimates, while photonics probability neurons (PPN) realized by the iMOCE supply statistically independent chaotic samples injected at layer activations. Repeating the forward pass for the same input yields Monte Carlo samples of a random function, from which both the predicted label and its epistemic uncertainty are extracted. As shown in Figure 6a, for a clear prediction, all predictive output samples collapse onto a single class, thereby enabling the activation probability of one output neuron to approach 1."

(11) Heart arrhythmia recognition acceleration:

What was the exact learning rule, rates, etc.? Reproducible for an external reader.

Response 11:

Thanks for the suggestion. We have added the detailed training parameters to the revised manuscript.

Specifically, the probabilistic neural network is structured as a 3-layer fully connected model. Each recorded ECG trace is converted into a 32-point vector via an FFT and fed to a hidden layer of 16 units. The output layer consists of 9 neurons. The network weights are pre-trained as point estimates. During

inference, physical randomness from the iMOCE is injected at the neuron activations through the modulator array. We perform 100 independent stochastic forward passes per input to obtain the activation distributions. ReLU activations and dropout layers are applied between layers. The final linear layer produces 9-class logits, from which we compute predictive entropy and mutual information to quantify uncertainty. Regarding the hyperparameters, Adam is employed as the optimizer, and the initial learning rate is set to 0.01 without decay. The batch size is 32, and the training duration is 200 epochs. The accuracy reported in the main text corresponds to the validation results after the 200th epoch.

Revisions:

The description has been added in the first paragraph, right column of page 8, as follows,

"Adam is employed as the optimizer, and the initial learning rate is set to 0.01 without decay. The batch size is set to 32, and the model is trained for 200 epochs. Notably, because the probability output of the integrated PPN depends on the bias applied to the MZM array, the statistical envelopes measured at the photodetector vary accordingly, as shown in Figure 6d."

(12) Heart arrhythmia recognition acceleration:

The acceleration through the iMOCE would also only apply to this case if the calculation of the BNN is bottlenecked by the creation of random numbers. Is this the case?

Response:

You are correct that for a standard digital BNN with limited sampling, the runtime is often limited by matrix multiplication rather than random number generation. However, the acceleration potential of the iMOCE becomes important in two specific large-scale probabilistic computing scenarios.

First, when the task requires lots of Monte Carlo sampling (hundreds of forward passes or thousands of agents), the generation of PRNG/TRNG and their on-chip distribution becomes a notable cost. Second, when probabilistic computing is pushed to the optical front-end or photonic tensor core, continuously obtaining randomness from the digital side brings I/O bandwidth and energy overhead. Using the same microcomb to supply both convolution carriers and Tb/s-rate physical entropy avoids the distribution bottleneck and supports scalable, low-latency operation. Such a situation has been shown in Figure R1 (To reviewer 1).

(13) Heart arrhythmia recognition acceleration:

The shown confusion matrix and data visualization are nice, but they represent a property of the BNN and learning algorithms, not of the iMOCE? Or would the authors claim that the iMOCE provided any actual performance better than a different random number generator? I am explicitly not talking about efficiency. Is there any difference from white noise generated in any other way?

Response 13:

In the ECG recognition task, we use a moderate network and a 9-class arrhythmia dataset. We agree that the classification performance depends on the model architecture, loss, and training data rather than on the origin of the probability signal. But our goal is to claim that the iMOCE can act as a high-quality physical random source for probabilistic computing without degrading the performance of a realistic medical task.

To answer the reviewer's question more directly, we have added an experiment comparing different kinds of randomness sources. Specifically, we also study the PRNG and amplified spontaneous emission (ASE) noise sources in our experiment. The resulting learning curves are shown in Fig. 6, where three random sources achieve the final accuracy of 95.56, 94.6, and 92.59%. The performance of the iMOCE is statistically comparable to that of the high-quality PRNG, confirming that it serves as a robust substitute.

Therefore, the distinguishing benefits of the iMOCE are architectural rather than algorithmic. iMOCE can serve as a multichannel, high-rate entropy source that substitutes for a digital PRNG without sacrificing computing performance. Compared with conventional noise sources, iMOCE provides Tb/s-rate physical entropy, can share the same microcomb used for convolution to tightly couple randomness with photonic operators, and is suitable for highly parallel, long-horizon workloads such as multi-agent RL and large-scale Monte Carlo tasks. These properties distinguish iMOCE from purely digital white noise and align with the needs of future probabilistic photonic computing.

Revisions:

The description has been added in Figure 6 and the third paragraph, right column of page 8, as follows,

"

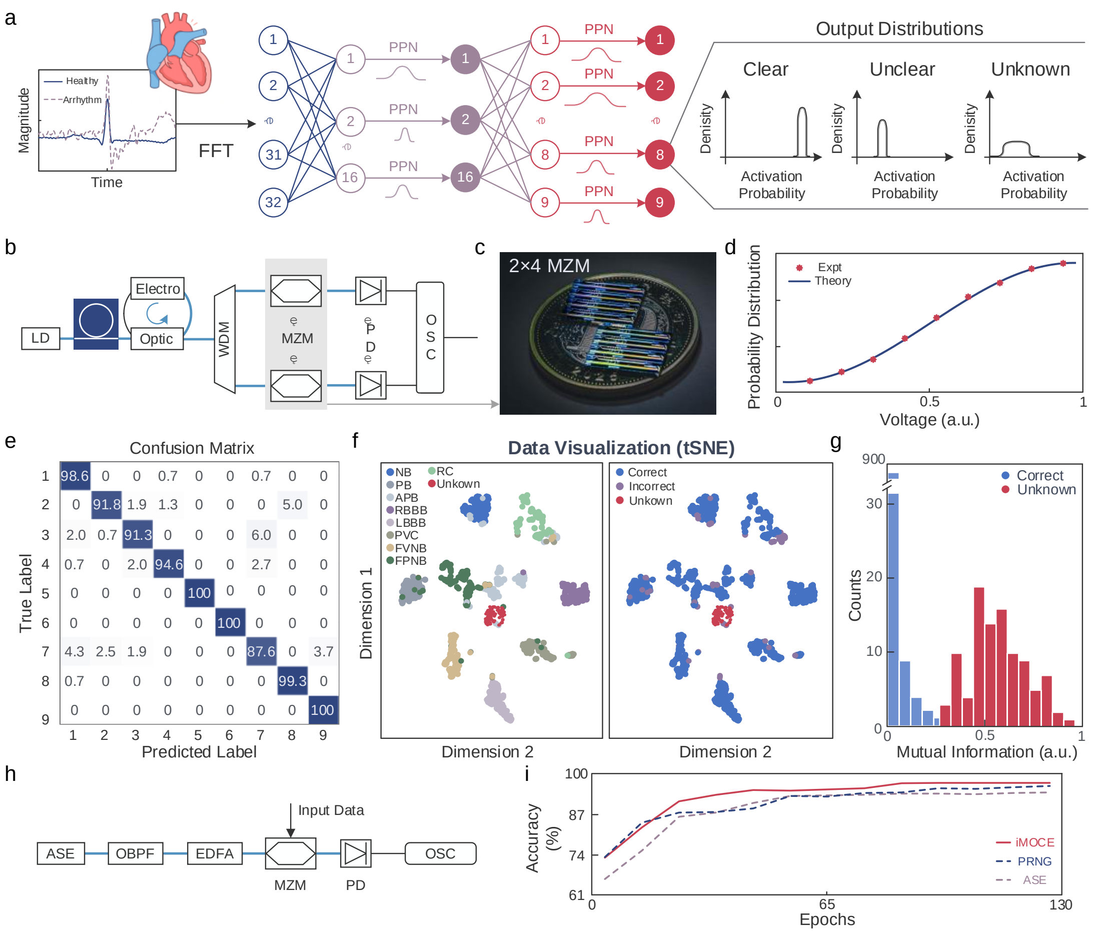

Figure 6 a. The setup of the heart arrhythmia recognition task acceleration based on the iMOCE. Each ECG trace is converted into a 32-point vector through a Fast-Fourier Transform (FFT) and fed to a two-layer probabilistic neural network. b. The schematic diagram of the photonics probability neuron (PPN). iMOCE supplies statistically independent chaotic samples through the network

weights to realize variational posterior sampling. After training, the network outputs a posterior class-probability vector per input, from which the predicted label and epistemic uncertainty are obtained. c. The microscope photo of the MZM array. d. The probability distribution of the generated chaos as the applied voltage is changed. e. The confusion matrix of the recognition model with a recognition accuracy of 95.7\%. f. The t-SNE visualization of the input data, where nearby points correspond to similar data and distant points to dissimilar data. g. The mutual information distribution of the correct and unseen data. Mutual-information distributions for correct versus unseen data show clear separation, demonstrating successful rejection of out-of-distribution inputs. h. Experimental setup of the amplified spontaneous emission (ASE) noise-based optical noise source. i. Validation accuracy as a function of training epochs when driven by three different random sources.

Finally, the mutual-information map in Figure 6g reveals a pronounced separation between correct and unknown samples, demonstrating successful rejection of OOD inputs.

To test the performance of the ECG task under different random sources, the probability training is repeated while the iMOCE chaos is replaced by either a digital pseudo-random number generator (PRNG) or an amplified spontaneous emission (ASE) noise-based optical white-noise source (Figure 6h). As shown in Figure 6i, all three random sources produce nearly identical learning curves and converge to final accuracy of 95.5%, 94.6%, and 92.3%, respectively. This confirms that the iMOCE acts as a high-quality physical random engine that can replace conventional noise sources without degrading accuracy."

(14) Multi-armed bandits (from the Supplementary):

The caption of Fig. 2a in the supplementary claims that in each cycle, the machine "with the highest biased matrix" is played. This should probably refer to the highest entry of the matrix? Otherwise, the authors need to explain how an entire matrix is evaluated as being "highest.

Response 14:

Thanks for the suggestion. In the multi-armed bandits, the machine i with the highest biased score $A_i(t)$ is played. In the revised manuscript, we have corrected the caption to avoid ambiguity.

Revisions:

The caption of Figure S1 has been revised, as follows,

"In each cycle, the slot machine i with the highest biased score $A_i(t)$ is played. This balances exploration and exploitation and supports efficient convergence under non-stationary rewards."

(15) Multi-armed bandits (from the Supplementary):

How the actual setting of the arms was created is important. Listing just a few entries is insufficient; instead, the authors should describe the process of assigning P_i.

Response 15:

Thanks for the suggestion. Each arm has a fixed but unknown reward probability P_i, which the player can only estimate through interaction during simulation. For all non-optimal arms, the reward probability P_i is independently selected from 0.3 to 0.8. For the optimal arms, the reward probability P_i is set to 0.9. The reward probability P_i is initialized once per independent simulation and kept constant throughout.

Revisions:

The description has been added in the second paragraph of supplementary page 3, as follows,

"For all non-optimal arms, the reward probability P_i is independently selected from 0.3 to 0.8. For the optimal arm, the reward probability P_i is set to 0.9. It's obvious that the iMOCE accelerator rapidly converges to the optimal arm."

(16) Multi-armed bandits (from the Supplementary):

Figure 2a shows a comparison between different MAB methods. The iMOCE is shown to solve the problems faster than other methods. This seems, quite frankly, a questionable claim. Don't the authors espouse the fact that the iMOCE produces very nice, symmetric chaotic signals that do not require complex post-processing? Then what special property should give these chaotic signals an edge in this task? Followingly, the authors should also compare whether the performance is identical to pseudo-random-number-generated white noise. I reiterate that it is not clear why iMOCE should have any better performance on this task - the iMOCE is supposed to supply random numbers for various applications, which it should do exactly as well as any other source.

Response 16:

Thanks for the suggestion. The reviewer is absolutely correct that the symmetric property of the iMOCE chaos primarily ensures high randomness quality and post-processing efficiency, rather than providing an inherent algorithmic acceleration per step compared to an ideal random source.

The convergence speed in the tug-of-war MAB algorithm is heavily dependent on the hyperparameter k, which controls the balance between exploration and exploitation [R5]. In prior works [R10-R12] focusing on smaller tasks (N=4-256), smaller k values are typically used. In our work, focusing on larger tasks (N=16-1024), we adopt a constant value k=0.08 that is chosen as a compromise across this range of N. As a result, our implementation of the same tug-of-war algorithm converges faster than those in references [R10-R12]. When we reduce the value of k, the required number of plays will increase, and the scaling becomes closer to previously reported results.

Moreover, we replace the chaos generated by the iMOCE with the PRNG and ASE noise in the revised manuscript. As shown in Supplement Figure 1e, their convergence cycles exhibit similar distributions. This indicates that, once k is fixed, the learning dynamics are essentially insensitive to the physical origin of the randomness, and iMOCE can be used as a high-quality replacement for conventional electronic and optical random-number sources.

[R5] Xu, Z., Tian, H., Zeng, Z. et al., Harnessing a nonlinear optoelectronic oscillator for speeding up reinforcement learning, *PhotoniX* 6, 5 (2025).

[R10] Shen, B., Shu, H., Xie, W. et al., Harnessing microcomb-based parallel chaos for random number generation and optical decision making, *Nature Communications* 14(1) (2023) 4590.

[R11] Morijiri, K., Mihana, T., Kanno, K. et al., Decision making for large-scale multi-armed bandit problems using bias control of chaotic temporal waveforms in semiconductor lasers, *Scientific Reports* 12 (2022) 8073.

[R12] Morijiri, K., Takehana, K., Mihana, T. et al., Parallel photonic accelerator for decision making using optical spatiotemporal chaos, *Optica* 10(3) (2023) 339-348.

Revisions:

(1) The description has been added in the second paragraph of supplementary page 7, as follows,

"where $X_i(t)$ and $B_i(t)$ represent the chaos sequence and value estimation, respectively. k is the value coefficient, which strongly influences the balance between exploration and exploitation. A smaller k keeps the dynamics closer to unbiased random exploration, requiring more plays to select the best arm. A larger k amplifies the current bias and accelerates exploitation, but risks locking onto a sub-optimal arm. In this problem, k is set to 0.08."

(2) The description has been added in Figure S2 and the third paragraph of supplementary page 4, as follows,

"

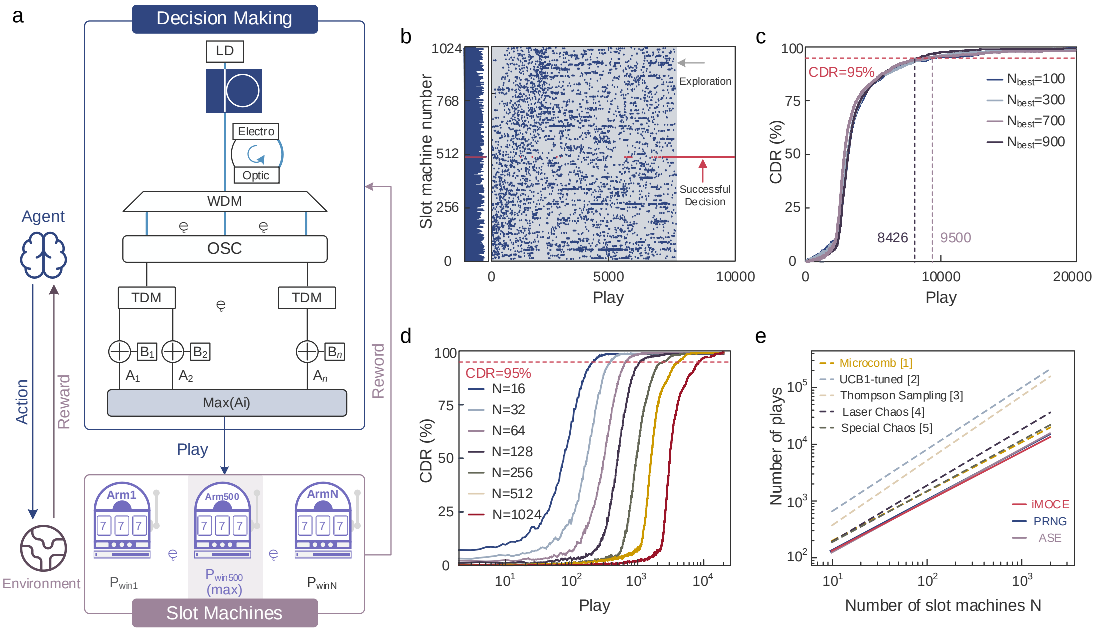

Figure S2 a. The setup of the MAB problem based on the iMOCE. Parallel chaotic channels are time-division multiplexed into multiple sub-series, each mapped to one arm. The generated chaos matrix is combined with the value matrix to compose the bias matrix. In each cycle, the slot machine i with the highest biased score $A_i(t)$ is played. This balances exploration and exploitation and supports efficient convergence under non-stationary rewards. b. The decision process of the 1024-armed MAB problem, where the left figure indicates the initial probability distribution of the slot machines. c. The trend of the corrected decision rate (CDR) with the increase of plays, where the red dotted line marks the CDR of 95%. d. The trend of the CDR under different scales. e. The comparison between the iMOCE and other optical chaotic sources. The iMOCE accelerator achieves faster convergence with fewer explorations, locating the optimal arm more rapidly and improving overall performance. The curves also compare the iMOCE with a digital PRNG and optical ASE noise. The overlapping results confirm that iMOCE achieves equivalent algorithmic performance to standard high-quality random sources.

Consequently, the iMOCE accelerator locates the optimal arm more rapidly, substantially enhancing the overall system performance. To further distinguish the role of the random source from that of the tug-of-war update, the MAB tasks are repeated using a digital pseudo-random number generator (PRNG), and optical amplified-spontaneous-emission (ASE) noise shaped by the setup in Figure 2h. As shown in Figure S2e, the three curves almost overlap and follow a similar power-law scaling with N. These results confirm that iMOCE provides randomness that is equivalent to high-quality PRNG and ASE noise for

this task, while offering much higher physical throughput and scalability."

(17) Multi-armed bandits (from the Supplementary):

The authors must give the exact settings and formulas used for the other methods listed in Figure 2 a e - there are several hyperparameters for some of them.

Response 17:

Thanks for the suggestion. We have explicitly provided the selection rules and parameter settings for all algorithms presented in Figure S2e in the revised manuscript.

Revisions:

The description has been added in the second paragraph of page 4, as follows,

"For the microcomb, laser chaos, and special chaos system, it follows the same selection rules (tug-of-war algorithm) as the iMOCE system, while the chaos is generated by a microcomb, chaotic laser, or an OENC [1, 4, 5]. The relationship between the convergence period and the number of slot machines is $N_{CC}=26.48\cdot N^{0.89}$, $N_{CC}=22\cdot N^{0.98}$, and $N_{CC}=30\cdot N^{0.86}$, respectively. For the UCB1 algorithm, it follows the standard formulation listed in reference [2]. At play t, letting $\mu_i(t)$ be the empirical mean reward of arm i and $n_i(t)$ the number of times arm i has been played, the chosen arm can be expressed as

$$i_t = \arg\max_i \left[\mu_i(t) + \sqrt{\frac{2\ln t}{n_i(t)}} \right] \quad (3)$$

The relationship between the convergence period and the number of slot machines is $N_{CC}=59.2\cdot N^{1.08}$. The Thompson sampling algorithm follows the standard formulation listed in reference [3]. Rewards are modeled as Bernoulli variables. For each arm, a Beta posterior Beta(α_i,β_i) is maintained with a uniform prior $\alpha_i=\beta_i=1$. At play t, the chosen arm can be expressed as

$$i_t = \arg\max_i \theta_i^t \quad (4)$$

where θ_i^t is drawn from Beta(α_i,β_i). After observing the reward r_t, the posterior is updated by setting $\alpha_{i\_t}+r_t$ to α_{i_t}, and $\beta_{i\_t}+1-r_t$ to β_{i_t}, respectively. The relationship between the convergence period and the number of slot machines is $N_{CC}=30N^{1.13}$. As compared favorably with published approaches, the proposed scheme demonstrates faster convergence and fewer required explorations."

(18) Multi-armed bandits (from the Supplementary):

Did the authors optimize their own hyperparameters for the specific settings of P_i? If they compare their own MAB algorithm, which they optimized for their specific distribution of P_i, it will naturally lead to a better performance compared to another method that was optimized for a different P_i drawing. For example, the distance between the best and second-best arm, or the average win probability, etc. Figure 2 a e should not represent an unfair comparison. It seems likely that k=0.08 just happens to best match the P_i distribution shown, and not a general MAB case.

Response 18:

Thanks for the suggestion. We would like to clarify that we do not optimize the hyperparameter k for one specific realization of the reward probabilities P_i. Instead, k=0.08 is chosen as a robust constant suitable for the general class of large-scale MAB tasks (N=16-1024) targeted in this work.

As shown in our Response 15, the reward probability P_i is independently selected from 0.3 to 0.8. For the optimal arms, the reward probability P_i is set to 0.9. The P_i used in Supplementary Figure 2b is randomly drawn from the same family, and the best arm and the distance between the best and second-best arm are also changed. In this situation, the iMOCE system can also select the best arms within this distribution family.

Revisions:

The description of the hyperparameter has been added as detailed in our Response 16.

(19) Multi-armed bandits (from the Supplementary):

Once again, the technical details are lacking - were the matrices A and B actually implemented on chip? Max(A_i) was implemented how? Could the oscilloscope actually read all channels simultaneously, or was this assembled digitally later, etc.?

Response 19:

iMOCE generates the on-chip multichannel physical random sequence $X_i(t)$, and the matrices A and B are computed digitally from the sampled data instead of being operated on the chip.

Because the Keysight UXR3304B is a four-channel real-time oscilloscope, for larger-arm bandit settings, we follow the scheme in reference [R5]. We use the time-division multiplexing method, where long records from multiple chaotic channels are partitioned into sub-sequences and then processed.

[R5] Xu, Z., Tian, H., Zeng, Z. et al., Harnessing a nonlinear optoelectronic oscillator for speeding up reinforcement learning, *PhotoniX* 6, 5 (2025).

Revisions:

The description has been added in the second paragraph of supplementary page 2 as follows,

"The goal is to identify the slot machine with the highest P_i through iterative exploration. During the exploration process, the parallel chaotic channels are time-division multiplexed to form N sub-series, with each sub-series representing one slot machine."

(20) Traveling salesman problem:

Here, the iMOCE acts as the noise term for an annealing problem, as far as I can tell. The method is described only very briefly, so it is difficult to understand what exactly has been done.

Response 20:

The reviewer is absolutely correct that the iMOCE here acts as a stochastic noise term to enable an annealing-like process. The description is indeed brief in the original text, and we have expanded it in

Supplementary Note 3 to provide precise mathematical details.

Specifically, at each iteration t, for each neuron (i, j), the state matrix is updated according to

$$s_{i,j} = \begin{cases} +1, & \text{if } WF_{i,j} + k_c X > \theta \\ -1, & \text{otherwise} \end{cases} \quad (3)$$

where $WF_{i,j}$ is the weight matrix, X is the chaotic sequence, θ is a fixed threshold, and k_c=1.5 is a scalar that controls the chaotic strength. In practice, X is supplied by the iMOCE. One optical chaos channel is photo-detected and continuously captured by a real-time oscilloscope, and the obtained samples are streamed to the Hopfield solver (one sample per neuron update step). When k_c=0, this reduces to a deterministic Hopfield network that often gets trapped in local minima. When k_c=1.5, the iMOCE term provides time-varying perturbations analogous to simulated annealing, enabling the network to escape minima and explore shorter tours.

Revisions:

(1) The description has been added in the first paragraph of supplementary page 6, as follows,

"where k_c=1.5 scales the influence of the chaotic fluctuation. In practice, X is supplied by the iMOCE. One optical chaos channel is photo-detected and continuously captured by a real-time oscilloscope, and the obtained samples are streamed to the Hopfield solver (one sample per neuron update step). When k_c=0, this reduces to a deterministic Hopfield network that often gets trapped in local minima. When k_c=1.5, the iMOCE term provides time-varying perturbations analogous to simulated annealing, enabling the network to escape minima and explore shorter tours."

(2) The description of the external situation and settings has been added as detailed in our Response 21.

(21) Traveling salesman problem:

The caption of Figure (3a) b, that "without chaos," things get stuck in a local minimum, is not surprising; an annealing process does not work without a noise source. Once again, this is an application for noise, but it is not clear why this showcases anything in particular about the iMOCE, unless a significant part of this problem was actually hardware-accelerated. It seems the iMOCE once again only supplied random numbers. White noise created by a pseudo-random number generator should have the same effect?

Response 21:

Thanks for the suggestion. The comparison with the "no-chaos" case is introduced primarily to validate the experimental setup, rather than to claim a novel algorithmic discovery, as an annealing process inherently requires noise to escape local minima.

In addition, we also repeat the experiment by replacing the iMOCE signal with the PRNG and ASE noise signal and obtain nearly identical terminal-energy histograms and optimal-tour success rates, as shown in Figure S3. This confirms that, for this task, iMOCE serves as a high-throughput, high-quality physical perturbation source, while the Hopfield dynamics itself governs the optimization behavior. In the revised manuscript, we have softened the description and clarified that our contribution is to demonstrate iMOCE as a versatile physical randomness engine, not a new optimization algorithm.

Revisions:

The description has been added in Figure S3 and the fourth paragraph of supplementary page 6, as follows,

“

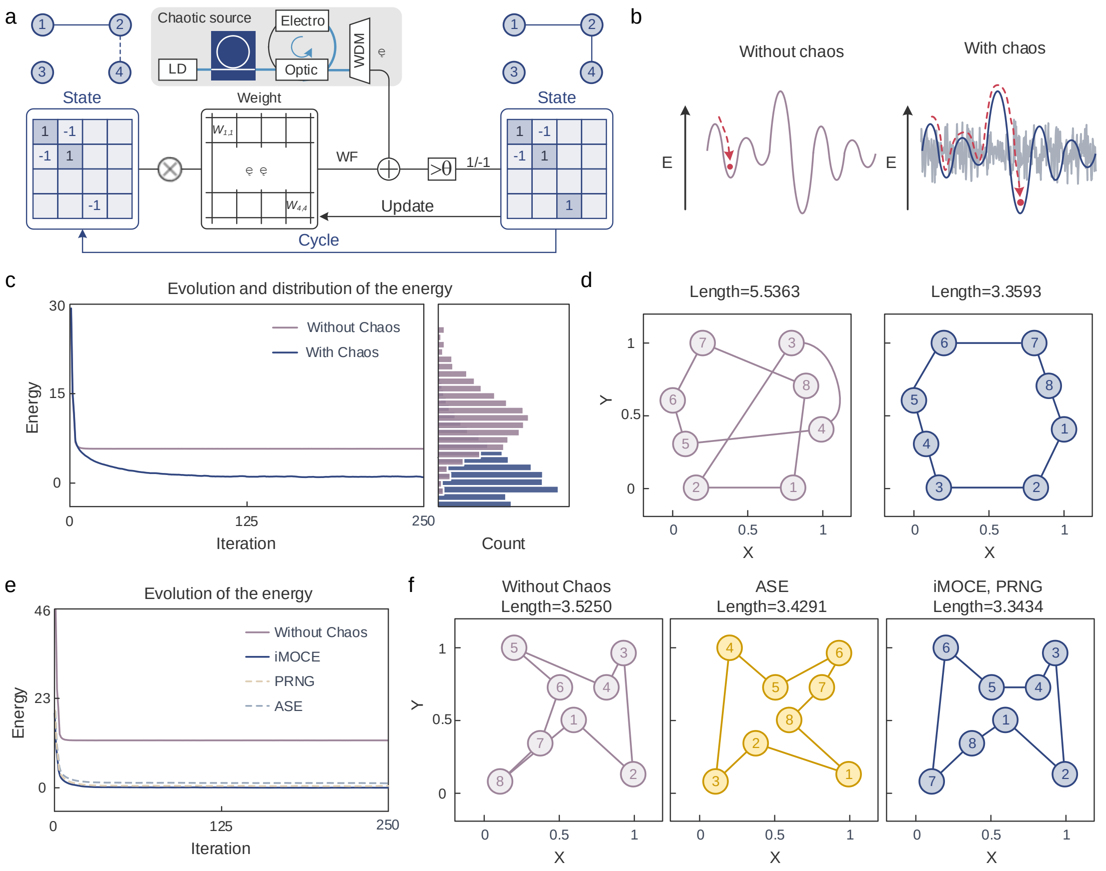

Figure S3 a. The setup of the traveling-salesman problem acceleration based on the iMOCE. b. The schematic depiction of the global minimization process enabled by chaos. Broadband perturbations from the iMOCE inject stochastic kicks that help the solver escape local minima and mitigate premature convergence. c. The evolution and distribution of the energy with/without chaos. Chaotic injection yields faster descent and a lower steady-state energy, with distributions concentrated near the global optimum. d. The results of the convergence route. With chaotic perturbations, the trajectory smooths and reaches the global optimum. Thus, chaos accelerates convergence and prevents suboptimal trapping. e. The evolution of the Hopfield-network energy for a second 8-city task under four perturbation conditions, including deterministic update without chaos, and stochastic updates driven by iMOCE chaos, electronic PRNG, and optical ASE noise, respectively. f. The best tours found for the second 8-city task.

In each trial, the constraint coefficients are fixed to C_1=2, C_2=2, C_3=0.9, and C_4=0.21. The threshold θ is set to 66. All 64 neurons are updated once per iteration, and the network is evolved for 250 iterations. Figure S3c indicates the trend curve of energy E over 1000 randomized instances of the traveling-salesman problem.

……

To verify that the observed effect is neither specific to one city layout nor unique to iMOCE, a second 8-city task is demonstrated. For this fixed graph, 1000 dependent trials of the Hopfield network under four conditions are tested, including without any perturbation, and with stochastic perturbations

provided by iMOCE, PRNG, and ASE noise. Figure S3e plots the average energy versus iteration. Without chaos, the dynamics quickly settle into a high-energy local minimum and remain trapped, whereas all three noise sources drive the network out of the minimum and towards the global optimum. The corresponding best tours are shown in Figure S3f, where the deterministic case yields a sub-optimal route of length 3.5250, ASE noise improves this to 3.4291, and both iMOCE and PRNG perturbations converge to an identical tour length of 3.3434. These results confirm that, for the Hopfield-TSP task, iMOCE acts as a broadband physical perturbation that is algorithmically equivalent to conventional white-noise sources, while offering orders-of-magnitude higher random-bit throughput. Further tuning the chaos intensity and bandwidth may yield additional learning speed and accuracy gains."

(22) Traveling salesman problem:

Was only one graph tried? The one shown in the image? The setting is lacking technical details. The text mentions 1000 random initializations, but the sizes, settings, etc., are not mentioned.

Response 22:

Thanks for the suggestion. In the traveling salesman problem, we focus on a single example with 8 cities, corresponding to the locations illustrated in Figure S3d. Their 2D coordinates are first normalized to the unit square, and the inter-city distance matrix D is computed from the Euclidean distance between the normalized coordinates. The fixed 8-city graph is used for all results in Figure S3c-d. The "1000 random initializations" refer to 1000 independent trials of the Hopfield network on the fixed graph. In each trial, we use an 8×8 state matrix and update the value for 250 plays. At every play, all 64 neurons are visited once in a random order, and their local field WF is already given in Eq. (7). The constraint coefficients are fixed to C_1=2, C_2=2, C_3=0.9, C_4=0.21, and the threshold θ is set to 66. We will add these details to the revised manuscript to make the TSP task fully reproducible.

To verify that the observed result is not specific to a single city configuration, we have added an experiment on a new city location, with the result shown in Figure R3. The network parameters are kept identical to those used in Figure S3c-d. The result indicates that the role of chaotic perturbation generated by iMOCE is robust over different TSP graphs, and not limited to the single example shown in the main text.

Revisions:

The description of the external situation and settings has been added as detailed in our Response 21.

(23) The word 'Reward' is misspelled as "Reword" in several figures.

Response 23:

The word "Reword" has been corrected in the revised manuscript.

We are grateful that our manuscript has been reviewed by two reviewers. We appreciate that the reviewers read it carefully, offered suggestions, and provided an overall positive evaluation. We have carefully considered every issue raised by the reviewers and made the necessary revisions to improve the manuscript. In this response, we address all concerns and comments in a point-to-point manner. The reviewers' comments are in black, our responses are in blue, and the revisions made in the revised manuscript are in red.

Reviewer #1

As mentioned previously, I think this work is interesting and impressive from a photonics perspective, and the results in Fig. 4 already demonstrate performance. However, I still share the skepticism of the other reviewer regarding the quality of the other demonstrations using their generated chaos.

While I thank the authors for taking the time to augment their demonstrations (Fig 6, S2, S3) with comparisons to other forms of randomness, I feel that the authors may have missed the point of the criticism of myself and the other reviewer. The demonstrations in Fig. 5, 6, S2, S3 are not experiments that show the compelling advantage of iMOCE over standard RNG approaches.

(1) For instance, results that happen to show iMOCE as 'best performing' compared to PRNG and ASE (such as Fig 6i, S2e, S3e) might result from subtle differences in the shape or magnitude of the probability distribution rather than the quality of the noise in the first place. A small-scale 8-node traveling salesman problem does not seem to be the sort of problem that is highly sensitive to the sort of correlation biases that could cause issues in PRNG and ASE. These problems do not seem to be hardware-accelerated to use the speed of iMOCE.

In contrast, an application showing that the speed or quality of their noise is better than PRNG would be compelling. For instance, the authors mention that PRNG/etc. "can introduce correlation biases and weaken coverage of the policy space". It would be compelling if iMOCE was shown to avoid these issues for an important application, or shown to compute very fast. However, I recognize that this may be difficult or expensive to show in an experiment, and again find the results in Fig 4 to be more than sufficient by itself as a demonstration.

Response 1:

We sincerely thank the reviewer for the insightful comments. We fully agree with the reviewer that the primary value of the demonstrated tasks (MAB, TSP, ECG) in the current manuscript is to validate that iMOCE functions as an integrated high-speed, parallel physical random number generator capable of replacing standard RNGs without compromising performance, rather than to claim an inherent algorithmic advantage in convergence speed for these specific small-scale problems. Therefore, these experiments are intended to verify the feasibility of the source across heterogeneous tasks.

Meanwhile, we acknowledge that while the iMOCE generates entropy at Tb/s rates, realizing this throughput benefit at the system level in these specific demonstrations is currently constrained by the bandwidth of the electronic detection and digital processing interfaces. To fully demonstrate these experimentally at system scale would require substantially greater resources and funding.

Therefore, we have strictly followed the reviewer's comments to refine our claims on the existing demonstrations (Fig. 5, 6, S2, S3). We have clarified the advantages of the iMOCE in terms of speed and scalability, and avoided overclaiming its algorithmic gains in small-scale tasks and refined the language

throughout the manuscript. These revisions more accurately position the iMOCE as a high-throughput, integrated hardware entropy source statistically equivalent to ideal random sources, fulfilling the "hardware replacement" role as identified by the reviewer.

Revisions:

(1) The description has been replaced in the first paragraph, right column of page 3, as follows,

"Notably, the chip, fabricated in a commercial foundry and compatible with wafer-scale production, extends the per-channel 6-dB bandwidth to 25 GHz (two orders of magnitude beyond prior microcomb schemes). The feasibility of the multi-channel large-bandwidth chaotic entropy sources is verified by benchmarking five representative tasks, including random bit generation, MAB, connect-3, traveling-salesman solving, and electrocardiogram (ECG) trace recognition. By integrating optical chaos on a wafer-scale integrated photonic platform, iMOCE functions as a robust computing resource that provides a scalable, massively parallel physical entropy source for heterogeneous ML workloads and is poised for system-level deployment."

(2) The description has been replaced in the second paragraph, left column of page 9, as follows,

"Each task demonstrates that iMOCE provides high-quality physical entropy comparable to ideal sources, while enabling distinct hardware advantages in throughput and integration. Table 2 compares these results with leading optical accelerators, highlighting the superior performance of the realized iMOCE architecture."

(3) The description has been replaced in Figure S2, as follows,

"~~The iMOCE accelerator achieves faster convergence with fewer explorations, locating the optimal arm more rapidly and improving overall performance.~~ The curves also compare the iMOCE with a digital PRNG and optical ASE noise. The overlapping results confirm that iMOCE achieves equivalent algorithmic performance to standard high-quality random sources."

(4) The description has been replaced in Figure S3, as follows,

"The results of the convergence route. With chaotic perturbations, the trajectory smooths and reaches the global optimum. Thus, the injection of physical chaos enables the system to escape local minima and prevents suboptimal trapping."

(2) It could be argued that the demonstrations past Fig 4 (apart from MAB, which shows an advantage over other photonic approaches and appears to be a standard benchmark for hardware accelerators) detract from the quality of the manuscript because they do not show a strong advantage over existing techniques. I will leave it to the editor to decide if they would prefer some of the content to be trimmed. For publication in Nature Communications, I would be satisfied if the authors were to take slightly more care with their language and claims regarding Figs. 5, 6, S2, and S3.

For example:

(a) "The utility and generality of the multi-channel large-bandwidth chaotic entropy sources are validated by benchmarking five representative tasks, including the[omit: grammar] random bit generation, MAB, connect-3, traveling-salesman solving, and electrocardiogram (ECG) trace recognition. By integrating optical chaos on a wafer-scale integrated photonic platform, iMOCE functions as a reproducible computing resource that delivers end-to-end gains on heterogeneous ML workloads and is poised for system-level deployment."

'utility and generality' being 'validated' is a stretch because it seems that the authors do not use the 'large-bandwidth' or 'multi-channel' nature of their device in the demonstrations. 'delivers end-to-end gains on heterogeneous ML workloads' is a stretch; I do not think the authors demonstrate gains over

existing techniques.

(b) As an example of a well-posed claim, I approve of the accuracy of the end of the heart arrhythmia section: “This confirms that the iMOCE acts as a high-quality physical random engine that can replace conventional noise sources without degrading accuracy."

Here, the authors claim exactly what they did.

Response 2:

We thank the reviewer for providing specific and constructive examples of our claims. Regarding the reviewer’s specific critique in example (a), we agree that phrases like “validated utility” and “delivers end-to-end gains” are overstatements. We have removed these claims and adopted the formulation praised by the reviewer in example (b) as the standard for all application descriptions in the revised manuscript.

Revisions:

The description has been replaced as detailed in our response to Response 1.

To Reviewer 2:

The authors have presented an updated version of their manuscript on a new proposal for high-speed generation of random numbers via microcombs and opto-electronic feedback for on-chip integration.

The main appeal of the paper remains in the physical setup and experimental demonstrations. The authors have presented some additional arguments, but there remain some doubts about the real-world usability of such a large quantity of random numbers. Generously speaking, this reflects the fact that the RNG side is ahead of potential other components needed for system-scale use, e.g., photonic neural networks that might profit from it. Therefore, the experimental side of the paper presents a sufficient advance to merit publication in my view.

On the demonstration side, I think the clarifications now added by the authors were very necessary. The original manuscript suffered from a lack of details, which would leave a casual reader with wrong impressions that the iMOCE itself is responsible for solving heart arrhythmia, traveling salesman, decision making et cetera. The new comparisons to PRNG and ASE correctly show that it is merely a valid replacement for alternative RNG sources, and therefore correctly shifts the focus of the paper to the hardware aspects.

Traces of this "overselling" remain, in my view. From a principled perspective, showing the passing of the NIST suite should be sufficient. I will admit that the expanded set of examples in the manuscript helps to illustrate how various forms of RNG can be used across different aspects of machine learning and optimization algorithms, and therefore does not demand any further changes.

Overall, the paper is now suitable for publication from my perspective.

I could not find any available code to review.

Response:

We thank the reviewer for the positive evaluation and for recommending the publication of our work. Regarding the comment on “traces of overselling,” we have further revised the manuscript (in line with Reviewer 1’s suggestions) to ensure the claims are appropriate. Meanwhile, we have now uploaded the source code to the Code Ocean platform, a compute capsule platform recommended by *Nature Communications*, where the compute capsule is currently configured for peer review access. Notably, our uploaded compute capsule is structured into the following core components across multiple problems, which directly reproduce the experimental results discussed in our manuscript.

A. TSP problem

The code begins by initializing the TSP coordinates and formulating the baseline constraints for the neural network. It then implements the perturbation mechanism central to our approach. Notably, the chaotic sequence is generated algorithmically to ensure reproducibility within the Code Ocean digital environment. However, in practical real-world hardware, this module is connected directly with an OSA to acquire externally generated physical chaos signals. Finally, the code automatically logs the energy descent trajectories into CSV files and outputs the energy evolution and the optimized route figures.

B. MAB problem

The code first initializes the simulation environment with a specified number of parallel arms and assigns predefined reward probabilities. During the decision cycles, the algorithm dynamically calculates a bias matrix based on the real-time win/loss history of each arm and chaotic matrix. Finally, the

simulation automatically exports the continuous action selection history and probability distributions into CSV files.

C. Connect3 problem

The code establishes the game environment and initializes two competing RL agents that continuously update their state-value functions through thousands of repeated trials. Finally, the simulation automatically generates the win and draw rates of the agent and outputs learning curves to visualize the training evolution.
